# Randomized, Controlled Trial of Therapy Interruption in Chronic HIV-1 Infection

**DOI:** 10.1371/journal.pmed.0010064

**Published:** 2004-12-28

**Authors:** Emmanouil Papasavvas, Jay R Kostman, Karam Mounzer, Robert M Grant, Robert Gross, Cele Gallo, Livio Azzoni, Andrea Foulkes, Brian Thiel, Maxwell Pistilli, Agnieszka Mackiewicz, Jane Shull, Luis J Montaner

**Affiliations:** 1The Wistar Institute, PhiladelphiaPennsylvaniaUnited States of America; **2**Philadelphia Field Initiating Group for HIV-1 Trials and the Division of Infectious Diseases, University of PennsylvaniaPhiladelphia, PennsylvaniaUnited States of America; **3**Philadelphia Field Initiating Group for HIV-1 Trials, PhiladelphiaPennsylvaniaUnited States of America; **4**The Gladstone Institute of Virology and Immunology, University of CaliforniaSan Francisco, CaliforniaUnited States of America; **5**Center for Clinical Epidemiology and Biostatistics and the Division of Infectious Diseases, University of PennsylvaniaPhiladelphia, PennsylvaniaUnited States of America; **6**Department of Biostatistics, University of PennsylvaniaPhiladelphia, PennsylvaniaUnited States of America; University of OxfordUnited Kingdom

## Abstract

**Background:**

Approaches to limiting exposure to antiretroviral therapy (ART) drugs are an active area of HIV therapy research. Here we present longitudinal follow-up of a randomized, open-label, single-center study of the immune, viral, and safety outcomes of structured therapy interruptions (TIs) in patients with chronically suppressed HIV-1 infection as compared to equal follow-up of patients on continuous therapy and including a final therapy interruption in both arms.

**Methods and Findings:**

Forty-two chronically HIV-infected patients on suppressive ART with CD4 counts higher than 400 were randomized 1:1 to either (1) three successive fixed TIs of 2, 4, and 6 wk, with intervening resumption of therapy with resuppression for 4 wk before subsequent interruption, or (2) 40 wk of continuous therapy, with a final open-ended TI in both treatment groups. Main outcome was analysis of the time to viral rebound (>5,000 copies/ml) during the open-ended TI. Secondary outcomes included study-defined safety criteria, viral resistance, therapy failure, and retention of immune reconstitution.

There was no difference between the groups in time to viral rebound during the open-ended TI (continuous therapy/single TI, median [interquartile range] = 4 [1–8] wk, *n* = 21; repeated TI, median [interquartile range] = 5 [4–8] wk, *n* = 21; *p* = 0.36). No differences in study-related adverse events, viral set point at 12 or 20 wk of open-ended interruption, viral resistance or therapy failure, retention of CD4 T cell numbers on ART, or retention of lymphoproliferative recall antigen responses were noted between groups. Importantly, resistance detected shortly after initial viremia following the open-ended TI did not result in a lack of resuppression to less than 50 copies/ml after reinitiation of the same drug regimen.

**Conclusion:**

Cycles of 2- to 6-wk time-fixed TIs in patients with suppressed HIV infection failed to confer a clinically significant benefit with regard to viral suppression off ART. Also, secondary analysis showed no difference between the two strategies in terms of safety, retention of immune reconstitution, and clinical therapy failure. Based on these findings, we suggest that further clinical research on the long-term consequences of TI strategies to decrease drug exposure is warranted.

## Abstract 2

Structured treatment interruptions, or "drug holidays", in HIV patients yield no clinical benefits but don't lead to short- term treatment failure either

## Introduction

Antiretroviral therapy (ART) has been a milestone in the treatment of HIV infection. Current treatment guidelines for HIV-1 infection in the United States recommend the initiation of ART in patients with CD4 T cell counts of less than 350 cells/μl [1]. In implementing these guidelines, health-care providers face the ongoing challenge of developing treatment strategies that minimize drug-related toxicity and adverse effects while retaining effective control of viral replication. Furthermore, treatment-associated costs (particularly in resource-poor areas), difficulty in maintaining long-term optimal adherence [2], and the emergence of viral resistance [3,4,5] have limited the feasibility of life-long ART-mediated viral suppression, increasing the need for alternative treatment strategies. Intermittent therapy strategies, consisting of alternating cycles on and off ART, have increasingly emerged as a potential intervention to address limitations of continuous ART [6,7,8,9]. Therapy interruption (TI) studies in ART-treated patients with suppressed HIV infection [10] have addressed the general questions as to whether such strategies can achieve greater viral control through increased antiviral responses (autoimmunization hypothesis) or simply serve as a strategy to reduce cost of long-term therapy and drug-associated toxicity. While pilot studies and uncontrolled (or incomplete) trials in patients with chronic HIV infection have addressed viral and immune outcomes of fixed-length TI and fixed on-drug cycles [11,12,13,14,15,16], no completed randomized, controlled trial has yet addressed by intent-to-treat analysis the outcome during an open-ended TI of sequential TIs versus continuous treatment in patients with confirmed suppression. The largest study to date in this area is the prospective single-arm Swiss–Spanish Intermittent Trial (SSITT) conducted in 133 recruited patients undergoing sequential 2-wk TIs and showing a lack of impact of this strategy on achieving sustained viral loads of less than 5,000 copies/ml off therapy in those that completed the study [11]. However, the lack of a control arm in this study has left unanswered questions about the impact of multiple TIs on time to rebound, immune reconstitution, therapy failure, and viral resistance when analyzed against a randomized control arm of continuous treatment followed for equal time before a single open-ended interruption.

We completed a randomized, controlled trial on the outcome of repeated 2- to 6-wk TIs in patients with chronic infection in which the comparator group maintained continuous therapy and then an open-ended interruption period was applied in both treatment groups. The study addressed the potential for repeated interruptions of therapy to delay time to viral rebound as a primary outcome and analyzed secondary outcomes regarding study-defined safety criteria, viral suppression and resistance, and retention of immune reconstitution.

## Methods

### Participants

Between August 2000 and December 2003, we enrolled 42 patients infected with HIV who were older than 18 y and on ART; eligibility criteria included CD4 counts of greater than 400 cells/μl on ART with a nadir of no less than 100 cells/μl, ART-mediated suppression (< 500 copies/ml) for more than 6 mo and less than 50 copies/ml at recruitment on any antiretroviral regimen. Approval of the study protocol was obtained from the institutional review board (IRB) of the Philadelphia Field Initiating Group for HIV Trials (Philadelphia, Pennsylvania, United States). Written informed consent was obtained from all patients. Human experimentation guidelines of the United States Department of Health and Human Services and of the authors' institutions were followed. The study protocol, including the patient consent form, the CONSORT form, and the IRB approval, can be found in Protocols S1–S4.

### Randomization and Study Design

Forty-two eligible patients from the Jonathan Lax Immune Disorder Clinic in Philadelphia, Pennsylvania, were randomized via sealed envelopes in a 1:1 fashion to a first phase (phase I) of either (1) three successive TIs of 2, 4, and 6 wk, respectively, or (2) maintenance of ART for 40 wk before a final interruption of therapy in both arms (phase II) subject to therapy reinitiation criteria as described below. Phase II consisted of an open-ended interruption to allow for virological and immunological comparisons between the groups off therapy. Study visits were every 2 wk for the repeated interruptions group and every 4 wk for the continuous ART group during phase I. Both groups were followed every 2 wk during phase II. We followed a study design with step-wise increases in the length of TI cycles to address potential safety concerns (resuppression was confirmed after shorter TIs before longer interruptions were initiated) and the hypothesis that sequential viral replication intervals would stimulate viral control and a delay in time to viral rebound.

Phase I procedures for the repeated interruptions group included the following. (1) Interruption of therapy was individually timed to occur after two HIV RNA measurements of less than 50 copies/ml without any viral load measurements greater than 400 copies/ml in between; these interruptions increased from 2 to 4 to 6 wk sequentially. (2) If a 0.5-log or greater reduction in viral load did not occur by 6 wk of reinitiated therapy or less than 50 copies/ml was not achieved within 20 wk of reinitiated therapy, patients were withdrawn as therapy failures and a resistance test was performed. (3) Patients were also withdrawn as therapy failures if (a) the CD4 cell number declined by more than 45% of the baseline CD4 count, (b) participants developed an opportunistic infection, even if retaining required CD4 count levels, or (c) a viral load of greater than 500,000 copies/ml occurred once, with or without development of acute retroviral syndrome as defined by fever, skin lesions, and pharyngitis.

Phase I procedures for the continuous therapy arm included the following: (1) patient monitoring if detected viremia was between 50 and 999 copies/ml, with the patient withdrawn if their viral load did not return to less than 50 copies/ml immediately prior to phase II, and (2) patient study withdrawal as therapy failure if during the 40-wk ART period viral load rebounded to more than 1,000 copies/ml at two consecutive time points.

Phase II procedures for both arms included the following: (1) monitoring for patient study withdrawal criteria as described in phase I, (2) determining time to primary end point of a viral load greater than 5,000 copies/ml, (3) monitoring until the time of therapy reinitiation at a viral load greater than 30,000 copies/ml for three consecutive time points, and (4) after reinitiation of therapy, follow-up on therapy to confirm resuppression to less than 50 copies/ml at 6, 10, and 14 wk on therapy. Clinical and laboratory parameters (CD4 count and viral load) were monitored at each visit, and venous blood was collected for additional secondary outcomes during selected study visits.

In both phase I and II, participants taking non-nucleoside reverse-transcriptase inhibitors (NNRTIs) were instructed to stop them a day earlier than the remaining drugs in the regimen.

### Primary and Secondary Outcomes

The primary outcome was time to confirmed virological rebound during phase II. Rebound was defined as first time point with greater than 5,000 copies/ml. Viral replication magnitude as defined by mean HIV-1 plasma RNA area under the curve (AUC_HIV RNA_) was measured as a secondary outcome at weeks 12 and 20 of phase II based on reinitiation-of-therapy criteria outlined above.

Additional secondary outcomes included (1) safety outcomes (serious adverse events [SAEs] and patient withdrawal based on criteria defined above), (2) retention of ART-mediated immune reconstitution, and (3) detection of viral resistance. Retention of immune reconstitution was analyzed by (1) same-day whole blood flow-cytometry-based analysis of CD4 and CD8 T cells, including total and naïve (CD62 l/CD45RA) and memory (CD45RO) subsets as described [17], and (2) same-day recall response analysis of peripheral blood mononuclear cell lymphoproliferative responses to Candida albicans as described [17]. Viral resistance mutations were retrospectively analyzed on cryopreserved plasma samples by genotyping of first available sample with viral load greater than 100 copies/ml following each interruption using the TruGene Assay (Visible Genetics, Toronto, Canada) at the Gladstone Institute of Virology and Immunology (San Francisco, California, United States) as previously described [18,19].

### Sample Size

The sample size required was calculated using PS [20] software, and based on a type I error of 0.05, with 90% power, to detect a difference of 4 wk or more in time to viral rebound between arms. Eighteen patients per group resulted in sufficient power (18 for 90%, 13 for 80%) to determine a difference of 4 wk or greater between groups in time to rebound of virus during the open-ended interruption. Assuming a loss to follow-up of 15%, we targeted 21 patients per group, or 42 total.

### Statistical Analysis

The primary analysis was an intent-to-treat analysis in which dropouts were assigned a week 0 rebound time (e.g., maximum failure to delay rebound). In secondary analyses, these dropouts were excluded. The log-rank test was used to test the null hypothesis of no difference between arms in the number of weeks from initiation of the open-ended TI to reaching viral rebound as defined. Patients not reaching end point at 26 wk after the beginning of the open-ended TI were censored. Wilcoxon rank sum tests were used to compare baseline and week 0 of the open-ended interruption between groups. Wilcoxon signed rank tests were used to test for no change from baseline to week 0 of phase II. Finally, Wilcoxon rank sum tests were employed to test between groups for equality of the mean AUC_HIV RNA_ up to 12 and 20 wk. In all cases, a two-sided alpha level of 0.05 was used to define statistical significance. Unless otherwise stated, results are presented as median (interquartile range) in text and tables.

## Results

### Patient Flow and Discontinuations

**Figure 1:**
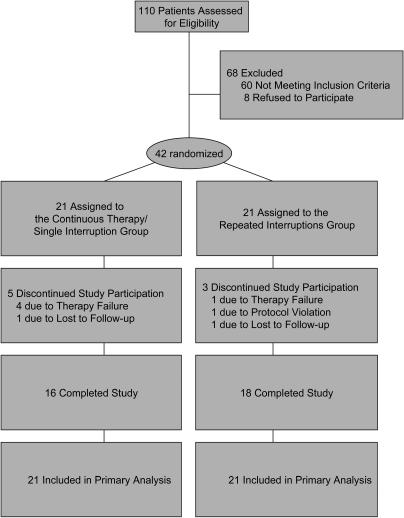
Study Flow

**Figure 2:**
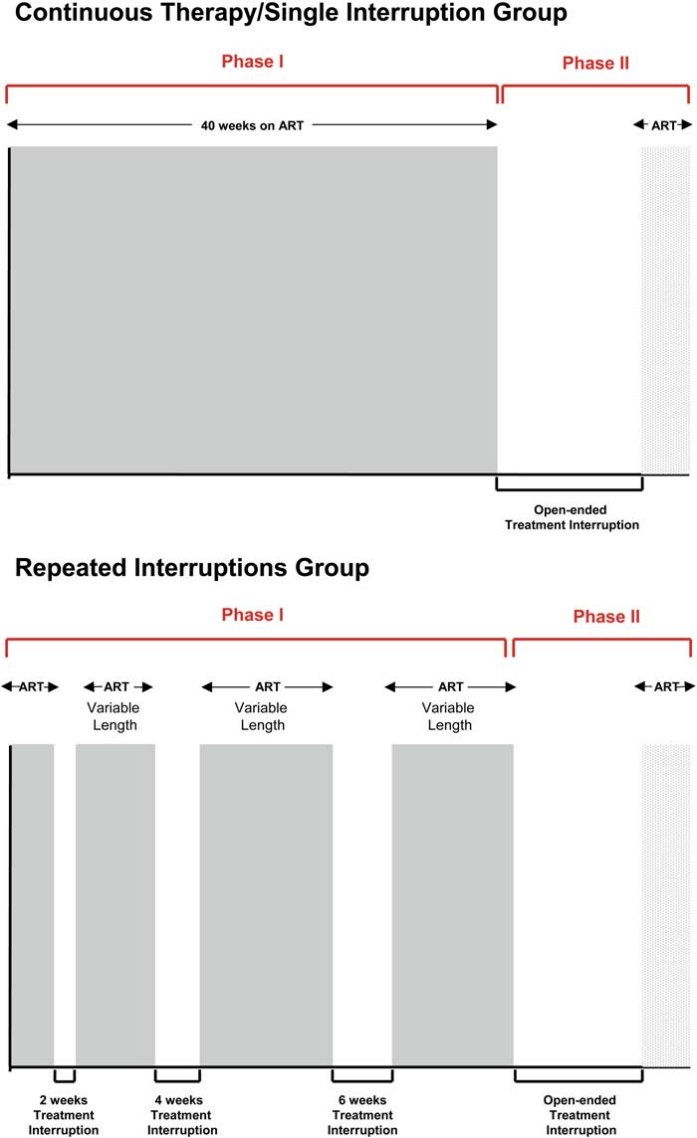
Study Design (Phases I and II)

Trial patient flow is summarized in Figure 1. Between August 2000 and December 2003, 42 patients at the Jonathan Lax Immune Disorder Clinic at the Philadelphia Field Initiating Group for HIV Trials were enrolled, randomized, and followed as shown in Figure 2. In the continuous therapy/single interruption arm, 16 of 21 patients reached the open-ended interruption. Reasons for study discontinuation in this arm were loss to follow-up (*n* = 1; patient moved away) and virological failure during continuous therapy (*n* = 4; further discussed below). In the repeated interruptions arm, 18 of 21 patients reached the open-ended interruption following three TIs of 2, 4, and 6 wk duration, with median peak rises in viral loads of 136 (50–2,590), 13,651 (180–222,589), and 18,887 (3,893–96,101) copies/ml, respectively. Median time to less than 50 copies/ml after resumption of therapy was 2 (0–4), 3 (1.8–12), and 9.5 (2–12) wk, respectively, with 9, 18, and 20 wk as the maximum time needed to achieve suppression in 100% of patients before reaching the open-ended interruption. Study discontinuation in the repeated interruptions arm was due to protocol violation (*n* = 1; patient restarted therapy during interruption out of protocol), loss to follow-up (*n* = 1; patient imprisoned), and virological failure during on-therapy period (*n* = 1; further discussed below).

### Baseline Criteria and Follow-Up

The demographic and clinical characteristics of the two groups at baseline are summarized in Table 1. Seventy-five percent of participants were on their second to fourth regimen while 25% were in their first regimen**.** No significant difference was found in baseline parameters between arms, with 33%–47% of patients on protease-inhibitor-containing and 61%–71% on NNRTI-containing regimens. Owing to the high participation of patients on NNRTI-based regimens and concerns about TI and safety in general, patient outcomes and treatment failure were reviewed monthly by the IRB of this study during the first 8 mo of study, quarterly for the following 4 mo, and semi-annually thereafter. Figure 2 shows study design for both arms, with a median follow-up of 41 (41–42) wk during phase I for the continuous therapy/single interruption arm and 42 (30–51) wk for the repeated interruptions arm. Follow-up during phase II had a median duration of 27 wk in both arms (continuous therapy/single interruption arm, 27 [8.75–47]; repeated interruptions arm, 27 [16.5–35]). Following reinitiation of therapy after phase II, patients suppressed viral replication to less than 50 copies/ml by a median time of 10 (6–12) wk in both arms, excluding for two patients in the continuous therapy/single interruption arm who elected to stay off ART indefinitely and one patient from the repeated interruptions arm who reported nonadherence following regimen reinitiation yet reached 52 copies/ml before withdrawing from additional follow-up.

**Table 1:**
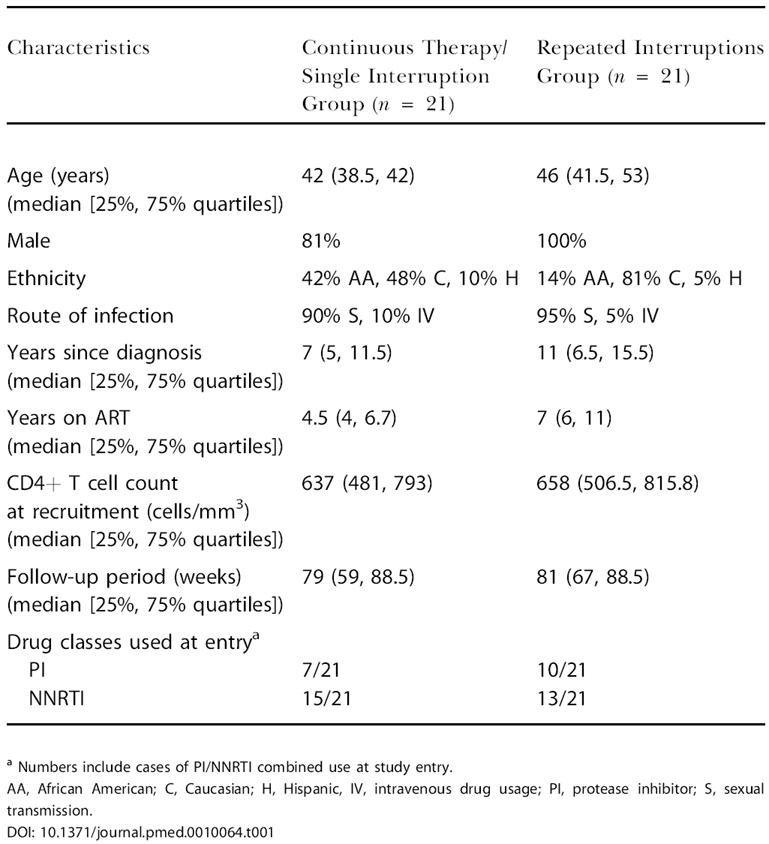
Baseline Demographic and Clinical Characteristics per Study Arm

^a^ Numbers include cases of PI/NNRTI combined use at study entry

AA, African American; C, Caucasian; H, Hispanic, IV, intravenous drug usage; PI, protease inhibitor; S, sexual transmission

### Primary Outcome

**Figure 3:**
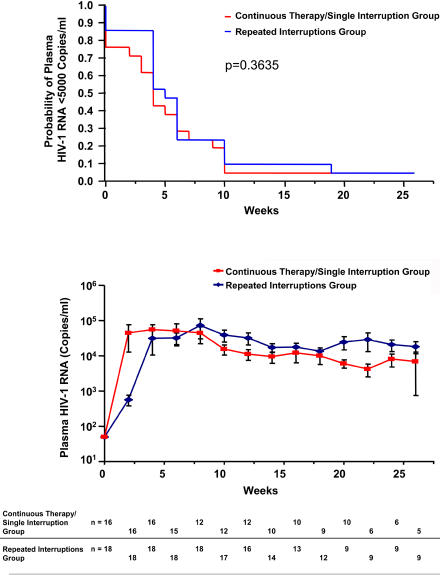
Lack of a Difference between Groups in Plasma HIV-1 RNA during Phase II Top panel shows Kaplan-Meyer plot summarizing time to a viral load of more than 5,000 copies/ml in both arms. Second panel shows viral load (mean ± standard error) per arm during 27 wk of TI (median time of phase II). Bottom table shows number of patients at time points shown for viral load in the second panel; the decrease in viral load over time is due to the reinitiation of therapy in patients with higher viral loads.

An intent-to-treat analysis of the time to viral rebound (>5,000 copies/ml) in the open-ended interruption showed no difference between groups (continuous therapy/single TI, median = 4 [1–8] wk, *n* = 21; repeated TI, median = 5 [4–8] wk, *n* = 21; *p =* 0.36). Figure 3 (top panel) shows the probability of plasma HIV-1 RNA remaining less than 5,000 copies/ml for the two groups (*n* = 21 per group). Exclusion of drop-outs in an as-treated analysis did not alter conclusions (single TI, median = 5 [4–9] wk, *n* = 18; repeated TI, median = 6 [4–8] wk, *n* = 16; *p* > 0.05). Additional secondary analysis of the magnitude of viral load as shown in Figure 3 (second panel) showed similar viral replication as determined by mean AUC_HIV RNA_ analysis at week 12 (single TI, median = 124,621 [23,326–262,348] AUC_HIV RNA_; repeated TI, median = 100,400 [47,221–365,731] AUC_HIV RNA_; *p* > 0.05) or week 20 (single TI, median = 114,550 [31,829–362,628] AUC_HIV RNA_; repeated TI, median = 153,097 [67,427–515,421] AUC_HIV RNA_; *p* > 0.05).

### Secondary Outcomes SAEs and patient discontinuation

No patient discontinuation in either group was due to study-defined changes in CD4 cell count (reviewed further below) or due to study-associated SAEs (disease progression or acute retroviral syndrome). However, four non-study-related SAEs occurred: two patients from the continuous therapy/single interruption arm were hospitalized, one for a cholecystectomy and one for acute rectal bleeding, during the 40-wk ART period; a patient from the repeated interruptions arm died of liver cancer during week 26 of the open-ended interruption after previously reaching a viral load greater than 5,000 copies/ml yet electing to stay off ART; and a patient from the repeated interruptions arm developed a transient ileitis.

### Immune reconstitution

**Figure 4:**
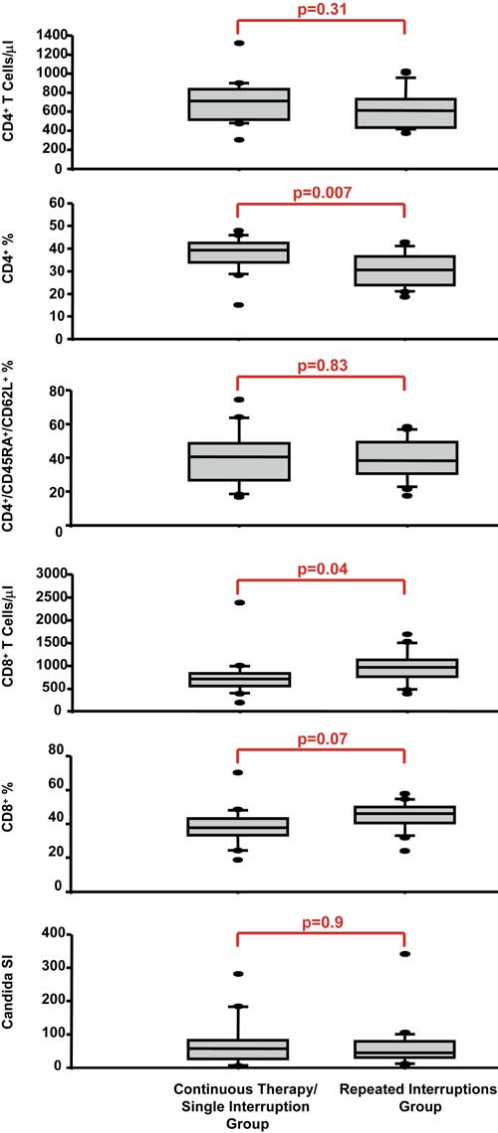
T Cell Subsets and Recall Lymphoproliferative Response at the End of Phase I End of phase I values for each arm are summarized (median and first and third quartiles) in the stacked figures showing from top to bottom: CD4 T cells/μl, CD4%, CD4^−^CD45RA^+^CD62L^+^% (naïve phenotype), CD8 T cells/μl, CD8%, and C. albicans lymphoproliferative response (shown as stimulation Index, SI). Unpaired *p* values for each variable are shown above corresponding bracket.

**Figure 5:**
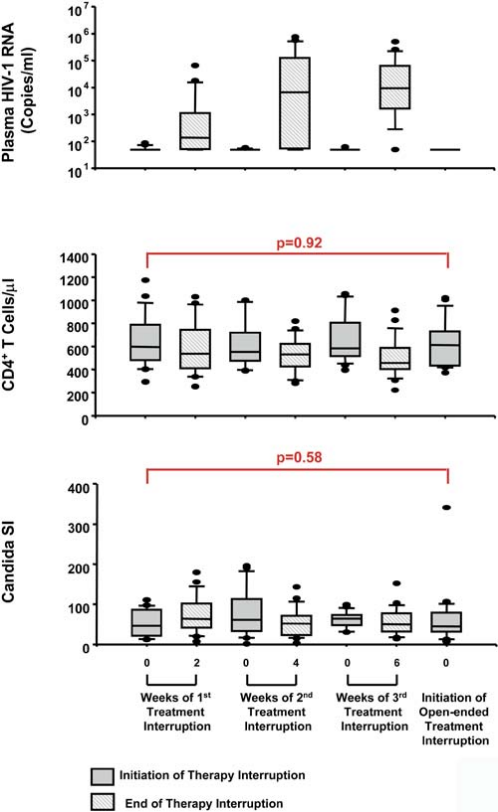
CD4 T Cells/μl and T Cell Recall Lymphoproliferative Response during Sequential TIs in Phase I Shown are data from the repeated interruptions arm. Panels show the TI initiation visit and TI end visit of each sequential TI inclusive of the initiation visit for phase II (open-ended TI).

No significant difference was observed between groups in CD4 T cell counts at the start of phase II, as illustrated in Figure 4. In addition, no difference in the percentage of naïve CD4 cells or decrease of recall response to C. albicans was observed, confirming the absence of significant differences in the retention of baseline immune reconstitution correlates between arms. However, a significant decrease in the abundance of CD4 cells relative to other T cell types as summarized in CD4% (but not in absolute CD4 count ) was present in the repeated TI arm, corresponding to a significant increase in CD8 T cell count. In spite of fluctuations in CD4 T cell count levels between the start and end of each monitored TI, a recovery of CD4 count levels was achieved upon resuppression following each TI in conjunction with a retention of lymphoproliferative responses against C. albicans before, during, and after each TI, as illustrated in Figure 5.

### Viral resistance mutations and therapy failure

An intent-to-treat analysis of the combined number of patients per arm with detected resistance mutations irrespective of therapy failure in phase I and during the final TI in phase II showed no significant difference between arms (continuous therapy/single TI, 7/21; repeated TI, 10/21; *p* > 0.05).

Study-defined criteria for therapy failure of a previously suppressive regimen were met by 4/21 patients in the continuous therapy/single interruption arm (patients S37, S47, S52, and S59) in association with self-reported nonadherence to therapy and detection of resistance mutations in phase I, as listed in Table 2. One patient in the repeated interruptions arm (1/21; patient S56) failed therapy after 20 wk following the third TI by maintaining a viral load between 50 and 999 copies/ml in the presence of previously undetected resistance mutations.

**Table 2:**
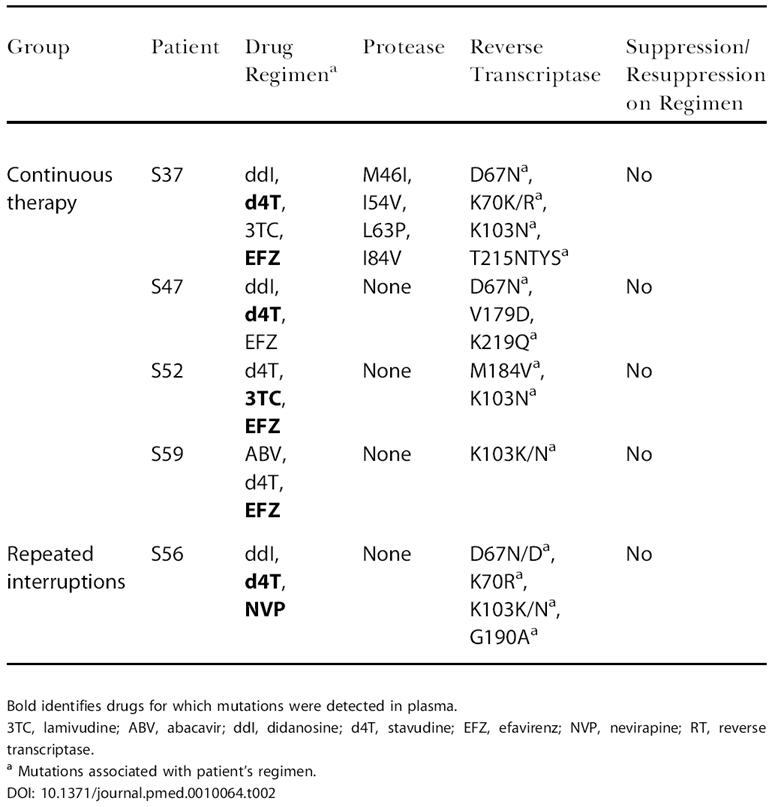
Therapy Failures with Plasma HIV-1 Protease and Reverse Transcriptase Inhibitor–Associated Resistance Patterns during on Therapy Periods (Study Phase I)

Bold identifies drugs for which mutations were detected in plasma

3TC, lamivudine; ABV, abacavir; ddI, didanosine; d4T, stavudine; EFZ, efavirenz; NVP, nevirapine; RT, reverse transcriptase

^a^ Mutations associated with patient's regimen

In patients who reached phase II in the absence of therapy failure, a total of 12 patients were identified to have resistance mutations at the first viremic time point (continuous therapy/single TI, 3/16; repeated TI, 9/18; *p =* 0.06). A greater number of resistance mutations was detected in the repeated interruption arm, as summarized in Table 3. In ten out of these 12 patients, a change in resistance patterns was observed when comparing the first viremic time point to the last. All 11 of 12 patients in Table 3 who reinitiated therapy retained suppressive ability of their respective regimens, as did all other patients who did not show resistance mutations in phase II. In the repeated interruptions arm, analysis of newly detected resistance mutations in phase II, as defined by a lack of detection during viremic time points in phase I, identified 3/18 patients (patients S4, S22, and S43) with this pattern (see notations in Table 3).

**Table 3:**
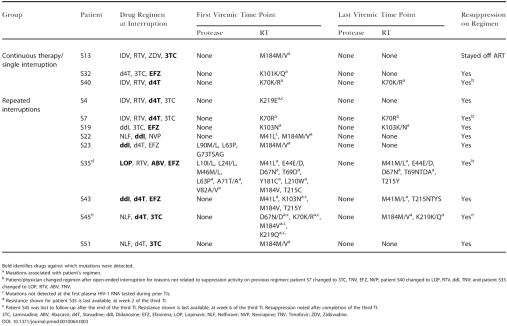
Non-Therapy Failures with Resistance Detected off ART at First and Last Viremic Time Point in Comparator Open-Ended TI (Phase II)

Bold identifies drugs against which mutations were detected

^a^ Mutations associated with patient's regimen

^b^ Patient/physician changed regimen after open-ended interruption for reasons not related to suppression activity on previous regimen: patient S7 changed to 3TC, TNV, EFZ, NVP; patient S40 changed to LOP, RTV, ddI, TNV; and patient S35 changed to LOP, RTV, ABV, TNV

^c^ Mutations not detected at the first plasma HIV-1 RNA tested during prior TIs

^d^ Resistance shown for patient S35 is last available, at week 2 of the third TI

^e^ Patient S45 was lost to follow-up after the end of the third TI. Resistance shown is last available, at week 6 of the third TI. Resuppression noted after completion of the third TI

3TC, Lamivudine; ABV, Abacavir; d4T, Stavudine; ddI, Didanosine; EFZ, Efavirenz; LOP, Lopinavir; NLF, Nelfinavir; NVP, Nevirapine; TNV, Tenofovir; ZDV, Zidovudine

## Discussion

Earlier reports on TI strategies in patients with chronic HIV infection include multiple pilot or single-arm study designs centered on the effects on viral control by comparison with pre-therapy periods, detection of resistance mutations without parallel follow-up of a continuously treated arm, and inclusion of variable criteria regarding viral resuppression before proceeding with repeated TIs [11,12,14,16]. In contrast, our strategy mandated resuppression of viral replication to less than 50 copies/ml before each TI and presents the first comparison of viral replication during a final open-ended interruption of therapy between patients randomized to complete three sequential TIs or stay under continuous therapy. Our data, based on intent-to-treat analysis, did not show that repeated TIs resulted in a clinically significant virological benefit as measured by the time to viral rebound to more than 5,000 copies/ml (see Figure 3). Secondary as-treated analysis on viral replication magnitude also indicated a lack of difference between arms. Consistent with the findings of SSITT [11], analysis of our data by the categorical classification of a “responder” as a patient with viral load less than 5,000 copies/ml at week 12 off therapy showed no significant difference in this frequency between arms (single TI, 5/18; repeated TI, 5/16), suggesting the presence of “responders” irrespective of previous protocol-mandated TIs.

Based on secondary outcome measures, the incidence of adverse events (SAEs, therapy failure, and patient discontinuation) or clinical disease progression (as indicated by CD4 count on therapy or opportunistic infections) was not observed to be different between arms. Prospective safety outcomes in our study are in accordance with reports from a retrospective analysis of 1,290 patients who interrupted treatment at least once (< 3 mo) without an increased risk of HIV-associated morbidity or mortality (with the exception of patients in Center for Disease Control and Prevention stage C during first interruption only) [21]. In regards to immunological outcomes, a concern associated with interruption of suppressive therapy is the potential for irreversible, viral-mediated CD4 T cell loss leading to disease progression [6,22]. We did not observe a decrease in CD4 cell numbers or lymphoproliferative responses against C. albicans when measured between arms before the open-ended TI (see Figure 4), nor following resuppression after monitored TI reinitiation cycles in the repeated interruptions arm (see Figure 5). The latter is consistent with observations by others and does not support an immediate immunological “cost” to short-term TIs [12,14,15,16,23]. However, we do show that monitoring CD4 cell numbers by percentage could lead to misinterpreting a significant loss of CD4 cells as a result of a significant increase in CD8 count following TIs, even though absolute CD4 count numbers remained unchanged (see Figure 4). Interestingly, the increase in CD8 T cell number also corresponded with an increase in HIV-specific responses as measured by interferon-gamma expression (data not shown), which in light of an absence of effect on viral load between arms further supports that TI strategies alone may not significantly alter the pre-existing balance between viral replication and host antiviral responses [14,16,23,24].

Importantly, no evidence for an increase of viral resistance in association with therapy failure was present in the repeated interruptions arm (See Table 2). We did not observe a greater clinical failure of NNRTI-based regimens in the repeated interruption arm due to “single drug” periods as predicted by recently redefined drug half-life estimates and the presence of viral replication during each interruption [25,26,27]. However, the percentage of patients with resistance mutations detected in this study in the repeated interruption arm (47%) is higher than the 17% observed in the SSITT cohort [11], in which patients with prior treatment failures were excluded [28]. We interpret this difference to mean that the resistance detected off drug in both our and their cohorts is likely associated with the greater number of drug-experienced patients in our cohort (75%) and the detection of prior archived resistance mutations as supported by Metzner et al. [29], who documented in 14/25 (56%) SSITT patients the presence of minor populations of M184V occurring at least once off drug during interruption of therapy.

In spite of the lack of difference in the total number of patients with resistant mutations detected on therapy during phase I and off therapy in phase II (7/21 [33%] versus 10/21 [47%], respectively) in both arms, we do report in similarity to others a greater detection of resistance mutations in the TI arm when restricting analysis to the last off-drug period only [29,30] as three of 16 (18%) had mutations detected off drug in the continuous therapy/single interruption arm compared to nine of 18 (50%) in the repeated interruption arm. However, based on the lack of association between viral resistance detected off-drug shortly after TI and resuppression by the same regimen in all patients, it remains undetermined to what extent TIs favor the detection of archived mutations in chronically suppressed patients and to what extent these mutations are a signal for a future therapy failure. The latter is best exemplified by the data we collected on patients on NNRTI-based regimens in the repeated interruptions arm where two patients (S19 and S43) showed K103N detection (only during the off-drug periods) in the absence of therapy failure while maintaining the same regimen after each TI, including post-study follow-up (Table S1). On the other hand, virological failure in the continued presence of an NNRTI-based regimen in phase I was associated with detection of K103N, as observed in one patient (S56) in the repeated interruption arm and three patients (S37, S52, and S59) in the continuous therapy arm with self-reported non-adherence.

Drug resistance that occurs during virological drug failure predicts virological responses to salvage treatment [31,32,33]. In contrast, the clinical implications of drug resistance mutations that appear shortly after TI in chronically suppressed patients are not clear. Case reports in this cohort of patients have demonstrated that drug-resistant variants that appeared during TIs may not persist in subsequent time points even after repeated use of the same antiretroviral regimen [19,34]. We now observe that drug resistance appearing during TIs can be transient since 50% and 33% of patients listed in Table 3 showed complete and partial reversion to wild type, respectively, when comparing to resistance at the last available viremic time point in phase II (See Table 3). Further, we observed durable resuppression of plasma viral RNA level in many patients who had drug-resistance mutations off therapy that would otherwise be expected to affect part of their treatment regimen when reinitiated (see Table S1). Virus populations that expand shortly after TI may lack all of the adaptations required to achieve high levels of plasma viremia in the presence of drug during continuous treatment. These adaptations may include the resistance-associated mutations, which were detected, as well as secondary mutations that may increase the viral replication capacity [35,36] or envelope adaptations required to escape concurrent humoral immune responses [37,38]. It is of interest to note that despite the large amount of research activity on TIs in patients with suppressed chronic infection and the hundreds of monitored interruptions studied to date, only limited cases of development of clinical resistance (as evidenced by a lack of viral resuppression following therapy reinitiation) have emerged, in contrast to the multiple reports of detection of viral sequences off ART associated with resistance as shown in this study and others [11,19,29,30,39,40].

Taken together, while our data show no clinically significant benefit for repeated TIs of less than 1.5 mo in patients with CD4 counts greater than 400 on therapy with regard to viral control as defined by time to rebound, secondary outcomes document no significant difference in levels of retention of immune reconstitution between arms and no increased incidence of virological failure as a consequence of TIs. While our data indicate that this TI strategy should not be pursued outside of a clinical trial setting, we argue that it will be important to collect additional data on the potential benefits of drug-sparing regimens (such as reduced long-term toxicity and reduced cost) and to define long-term outcomes in comparison with continuous therapy.

## Supporting Information

Registration of randomized trial at clinicaltrials.gov under identifier NCT00051818.

Protocol S1 Protocol Text: Effects of Sequential TI (614 KB DOC).

Protocol S2 Study IRB Approval Current IRB approval for study at clinical site.
(179 KB PDF).

Protocol S3 Wistar IRB Approval IRB approval to receive study biological material at the Wistar Institute for research.
(201 KB PDF).

Protocol S4 CONSORT Checklist (50 KB DOC).

Table S1 Patients with Detected Resistance during Phase II: Regimen at Initiation of Phase II and Subsequent Post-Study Follow-Up to August 2004 (36 KB DOC).

**Patient Summary**

**Why Was This Study Done?**

Highly active antiretroviral therapy has revolutionized HIV treatment for patients who have access to the medications. But the drugs are expensive, have side effects, and can become ineffective when the virus develops resistance. Structured treatment interruptions (STIs), also known as “drug holidays” (because patients take a holiday from their drugs), have been suggested as possible alternatives to continuous therapy. Initially, there was fear that patients who went back on therapy after an interruption would not be able to control the virus again, but there was also hope that STIs might actually strengthen the immune system. In addition, STIs might alleviate some side effects, and they would certainly reduce costs. This study uses a particular design to examine the risks and benefits of STIs.

**What Did the Researchers Do?**

The researchers studied 42 patients who received either continuous therapy for 40 weeks or three successive treatment interruptions of two, four, and six weeks, followed by a final open-ended interruption for both groups. The researchers then recorded how long patients were able to control the virus before their viral load reached a certain threshold and they had to restart therapy. They also examined CD4 counts and therapy failure, and looked for resistant viruses on and off therapy.

**What Did They Find?**

In terms of being able to control the virus, it made no difference whether patients were on continuous therapy or had three STIs. In other words, when both groups stopped treatment at 40 weeks, the length of time that the patients could control the virus was the same in both groups. Eventually, all patients (except two who elected to stay off antiretroviral therapy) re-initiated therapy because of a rising viral load, and the patients once on therapy all regained control over the virus. Resistant viruses were found in patients from both groups, but during the final interruption they were more common in the group that had received the three STIs.

**What Does This Mean?**

The study confirms that STIs do not help with viral control, consistent with other studies that found that STIs had no clinical benefit. On the other hand, no short-term adverse events were present, as all patients were able to regain control over the virus after they went back on treatment (without a drop in CD4 count), even after several rounds of interruptions and tests to detect of resistant viruses. There remains concern about whether recurrent cycles of viral replication and suppression might in themselves be harmful, and whether the presence of resistant virus is a signal for future treatment failure. Given these unanswered questions, STIs should only be undertaken within clinical trials.

**What Next?**

Possible risks and benefits of STIs in the management of therapy remain an active area of research. Evidence so far has not shown clinical benefits. Ongoing studies need to clarify whether there are long-term risks (and what they are), so that we can weigh these against the benefits of reducing costs and side effects.

**Additional Online Information**

The Body information Web page on STIs: http://www.thebody.com/treat/sti.html

Information on “continuing antriretroviral treatment” from AVERT, an international HIV and AIDS charity based in the United Kingdom: http://www.avert.org/conttrt.htm

Information on STIs from NAM, a United Kingdom registered charity: http://www.aidsmap.com/en/docs/7980314C-97B5–412F-93B1-AD8B64F51F73.asp

Factsheet on HIV treatments from the United States National Institute for Allergy and Infectious Diseases: http://www.niaid.nih.gov/factsheets/treat-hiv.htm

Search results from Clinicaltrials.gov when searching for “HIV” and “treatment interruption” combined terms: http://www.clinicaltrials.gov/search/term=%22Treatment+Interruption%22%5BCONDITION%5D+AND+HIV+%5BCONDITION%5D

## Abbreviations

ART: antiretroviral therapy
AUC_HIV RNA_: HIV-1 plasma area under the curve
IRB: institutional review board
NNRTI: non-nucleoside reverse-transcriptase inhibitor
SAE: serious adverse event
SSITT: Swiss–Spanish Intermittent Trial
TI: treatment interruption
