# Supplementary material for: Randomized, Controlled Trial of Therapy Interruption in Chronic HIV-1 Infection
Source: PLoS Med. 2004 Dec 28;1(3):e64. doi: 10.1371/journal.pmed.0010064 (PMC539050; doi:10.1371/journal.pmed.0010064)
Supplement: Protocol S1 — (614 KB DOC). [file pmed.0010064.sd001.doc]

# Title of Protocol

**Effects of Sequential Treatment Interruption: A single center, randomized, non-blinded study of the immune and viral outcomes of structured treatment interruptions (STIs) in chronically HIV-infected individuals on highly active anti-retroviral therapy (HAART)**

**Sponsored by:**

The Wistar Institute

Philadelphia FIGHT

**Support Provided by:**

The National Institute of Allergy and Infectious Diseases

Division of AIDS

(RO1 #AI48398)

**Principal Investigator:**

Luis J. Montaner, D.V.M., M.Sc., D.Phil.

The Wistar Institute

**Co-Principal Investigator (Principal Clinical Investigator):**

Jay R. Kostman, M.D.

Philadelphia FIGHT

**Co-Investigators:**

Cecile Gallo, B.S.N., R.N., Philadelphia FIGHT

Robert Grant, M.D., The Gladstone Institute of Virology and Immunology

Robert R. Gross, M.D., University of Pennsylvania

Michael McCune, M.D., The Gladstone Institute of Virology and Immunology

Karam Mounzer, M.D., Philadelphia FIGHT, Lax Treatment Center

Douglas Nixon,M.D., The Aaron Diamond AIDS Research Center

**Data Safety Monitoring Board::**

Julie Davids, B.S., Philadelphia FIGHT, Critical Path AIDS Project

Harvey Friedman, M.D., Chair, University of Pennsylvania

Ronda Goldfein, Esq., AIDS Law Project of Pennsylvania

Jay R. Kostman, M.D., Study Team Liason (non-voting member)

Rosalie Pepe, M.D., Episcopal Hospital

Version 1 (1/1/00)

Version 2 (6/6/00)

Version 3 (10/9/00)

TABLE OF CONTENTS

Page

STUDY SUMMARY……………………………………………………………………….… 4

1. STUDY OBJECTIVES……………………………………………………………………..6
   1. Primary Objectives
   2. Secondary Objectives
2. INTRODUCTION…………………………………………………………………………..7
   1. Background
   2. HIV-1 Therapy and Immune-Mediated Control
   3. HIV-1 Therapy and Toxicity
   4. HIV-1 Therapy and Adherence Monitoring
3. RATIONALE………………………………………………………………………………..11
4. STUDY DESIGN AND SAMPLE CALCULATION…………………………………..….12
5. SELECTION AND ENROLLMENT OF SUBJECTS…………………………………..…13
   1. Inclusion Criteria
   2. Exclusion Criteria
   3. Study Enrollment Procedures
      1. IRB Approval of Protocol and Informed Consent
      2. Informed Consent Process
6. STUDY TREATMENT……………………………………………………………………15
   1. Medication Regimens
      1. Stable HAART Therapy
         1. Nucleoside Reverse Transcriptase Inhibitors
         2. Non-Nucleoside Reverse Transcriptase Inhibitors
         3. Protease Inhibitors
      2. Dosing Adjustments
      3. Prohibited Medications
   2. Adherence Assessment
   3. Clinical Modulation of Medication
      1. Control Arm
      2. Experimental Arm
         1. Priming/Safety STI
         2. CD4 Boost STI
         3. CD8 Boost STI
         4. Comparison Treatment Interruption
         5. Six-Week Follow-Up
7. CLINICAL AND LABORATORY EVALUATIONS…………………………………….20
   1. Control Arm
   2. Experimental Arm
   3. Data Collection and Biological Samples
   4. Frequency of Sampling and Amount of Blood Drawn
   5. Study Discontinuation Procedures
8. CONCOMITANT THERAPY…………………………………………………………….27
9. ADVERSE EVENTS AND MANAGEMENT……………………………………………27
   1. Adverse Events
   2. Serious Adverse Events
   3. Pregnancies
   4. Study Discontinuation
10. CASE REPORT FORMS……………………………………………………………....29
    1. Source Documents
    2. Review of CRF Pages
    3. Changes in CRF Pages
    4. Data Verification
    5. CRF Completion
11. DATA COLLECTION…………………………………………………………………..30
12. SUBSTUDY DATA COLLECTION………………………………..……………….….30
    1. Data Collection for Antiviral CD4 T-Cell Responses
    2. Data Collection for Antiviral CD8 T-Cell Responses
       1. Cell Lines and ^51^Chromium Release Assays
       2. Tetrameric Peptide Complex Staining
       3. ELISPOT Assay
       4. Cytokine Flow Cytometry
    3. Data Collection for Cell Surface T-Cell Antigen Expression
       1. Time-Points
       2. Procedure
    4. Data Collection for Thymic Function
       1. Detection of T-Cell Receptor Excision Circles
       2. Time-Points
       3. Procedure
    5. Data Collection for Viral Genomes
       1. Rationale for Genotyping
       2. Rationale for Genotyping Assay Selection
       3. Time-Points
       4. Genotyping Assay Procedures
    6. Data Collection for Viral Reservoir
       1. Time-Points
       2. Procedures
13. STATISTICAL CONSIDERATIONS……………………………………………………39
    1. Efficacy Plan and Data Format
    2. Primary Analysis: Delay of Viral Rebound
    3. Secondary Analysis
       1. Recall responses: Antiviral & Candida
       2. Amplitude of Viral Rebound
       3. Analysis of Cell Surface T-Cell Antigen Expression
       4. Analysis of Thymic Function
       5. Analysis of Viral Genomic Data
       6. Analysis of Viral Reservoir Data
14. DATA SAFETY AND MONITORING BARD GUIDELINES………………………….45
    1. Main Safety Outcome End-Points
15. COMPENSATION, INSURANCE AND INDEMNITY………………………………….46
16. RETENTION OF RECORDS………………………………………………………………47
17. CONFIDENTIALITY OF INFORMATION………………………………………………47
18. REFERENCES………………………………………………………………………………48
19. ATTACHED APPENDICES………………………………………………………………56

###### SCHEMA

TITLE

**Effects of Sequential Treatment Interruption: A single center, randomized, non-blinded study of the immune and viral outcomes of structured treatment interruptions (STIs) in chronically HIV-infected individuals on highly active anti-retroviral therapy (HAART)**

DESIGN:

This proposal is centered on addressing immunologic and virologic outcomes in a single center, randomized, non-blinded study comparing a strategy of sequential structured treatment interruptions (STIs) versus continuous viral suppression. Forty-two subjects with CD4 >400 cells/mm^3^ with a nadir CD4 ≥100 cells/mm^3^ and having maintained viral suppression while on a Highly Active Antiretroviral Therapy (HAART) (VL < 500 copies/ml for 8 months and <50 copies/ml at recruitment) will be randomized in a 1:1 fashion to either (1) three successive treatment interruptions of 2, 4, and 6 weeks, respectively, or (2) maintenance of HAART. The successive interruptions will be timed so that they will be sequentially instituted the week after two consecutive HIV RNA measurements of < 50 copies/ml, measured two weeks apart. The primary analysis will be an intent to treat analysis of time to viral rebound (first time-point >5,000 sustained for three consecutive weeks) after a final period of a therapy interruption in both groups. Although safety will remain a primary focus of the analysis, the study is powered for the virological outcome rather than a safety outcome due to our preliminary safety data from monitoring periods without therapy that exceeded those used in this protocol. Safety will be assessed by changes in CD4 count, degree of viral rebound in response to treatment cessation, and symptomatic adverse events and will be monitored by a Safety Monitoring Board. Secondary immunological outcomes will be assessed by measurement of recall CD4 and CD8 responses (antiviral/Candida), cell surface antigen measurements of T-cell subsets and indirect measures of thymic output. Secondary virological outcomes will be measured by viral load, and viral genotyping at each rebound during STI. Overall, the proposed analysis is primarily centered on determining the safety, immunologic and virologic consequences of treatment interruption as a first step towards evaluating the role of this intervention in clinical management of chronic HIV-1 infection.

DURATION:

Subjects in the trial will be in the protocol for 61 weeks in the control arm and between 46-89 weeks in the experimental arm. The individualized progression through the protocol in the experimental arm as a consequence of sequence of events as defined by viral load results in a variable times & number of blood draws for each subject.

SAMPLE SIZE and POPULATION:

21 per arm will be recruited for a total of 42 subjects on this protocol. Patients will be recruited through Philadelphia FIGHT representing a network of clinics in Philadelphia (Jonathan Lax Immune Disorder Clinic, 800 patients; Infectious Disease Division at Presbyterian Medical Center, 350 patients; HIV Clinic at Temple University, 600 patients) providing clinical care to over 1700 HIV-infected persons.

The patient contact site will be the Jonathan Lax Immune Disorder Clinic. The patient community at this site is composed of 41% Female, 62% African American, 8% Hispanic with a predominance of 4-8 years of diagnosed HIV-1 infection and 25-45 years of age.

STRATIFICATION:

*N/A*

REGIMEN

N/A.

SUBSTUDIES

Anti-HIV Specific Immunity

Viral genotypes

Surface antigen expression in the T-cell subset

Thymic function assessment by T-cell excision circle measurements

*.*

STUDY OBJECTIVES

The long-range goal of this protocol is to determine if structured interruptions of Highly Active Anti-Retroviral Therapy (HAART) can be a safe and effective strategy to delay disease progression in chronically infected individuals. The short-term goal of this multidisciplinary collaborative team effort is to investigate the safety, immunologic, and virologic outcomes of sequential therapy interruption periods in chronically HIV-1 infected persons.

Over the past 14 months, analysis from an ongoing Wistar Institute observational study of chronically suppressed patients who interrupt therapy supports the working hypothesis that an acute viral rebound can boost anti-HIV-1 cellular immunity. Preliminary analysis of data collected every two weeks from the time of therapy withdrawal indicate a significant increase in antiviral CD4 T-helper and IFN-γ-secreting CD8 T cell responses. Specifically, viral re-bound was associated with a significant increase in anti-HIV-1 CD4 responses preceding a significant increase in CD8 response. Five untreated controls followed for a similar duration showed substantially fewer changes in immune response. Data from patients with high levels of anti-HIV-1 immunity at the time of therapy interruption or with more than one period of therapy interruption during follow-up suggests that boosted anti-HIV-1 specific responses can be associated with an increased control of plasma viral replication in the absence of therapy. To formally test the potential clinical relevance of these preliminary observations and due to community interest in recruitment into treatment interruption studies, this protocol will study the time to viral rebound after withdrawal of antiretroviral therapy in a well-defined subject population from a single center, in a randomized, non-blinded study where therapy adherence, and immune and viral outcomes will be monitored. The primary end-point of time to rebound and magnitude of viral response following treatment withdrawal will be compared between two groups: a group following a strategy of sequential structured treatment interruptions (STI) or one maintaining HAART therapy. Specifically, this study hypothesizes that repeated structured treatment interruptions in chronically suppressed patients will increase HIV-1 immunity and result in control of viral replication in the absence of therapy for a minimum of 4 weeks more than in controls who have remained suppressed.

To test this hypothesis this protocol will focus data collection and analysis on the following primary and secondary objectives:

# Primary Objective

- To determine if sequential STIs result in a period of 4 weeks or more of viral suppression in the absence of therapy when compared to an non-interrupted group maintaining continual suppression.

## 1.2 Secondary Objectives

- To evaluate the safety of sequential STIs.
- To determine if genotypic changes occur in the HIV-1 protease and reverse transcriptase regions after sequential STIs.
- To determine the effect of sequential STIs on recall responses compared to continuous HAART.
- To determine changes in T-cell activation, as measured by cell surface antigen changes, in relation to sequential STIs.
- To determine the role of thymic function on the response to sequential STIs.

# INTRODUCTION

## 2.1 Background

The recent realization that HIV-1 is continually replicating in spite of effective anti-retroviral therapy, the identified toxicities of long-term HAART, the recognized difficulty of long-term adherence to therapy, and the discovery that resting CD4 T-cells can provide a reservoir for HIV-1 with a predicted decay rate of about 60 years have reinforced the need to develop new approaches to control and treat HIV-1 infection 1-6. One area that is increasingly targeted for clinical development is the potential of immune-based therapies to complement existing anti-retroviral drugs 6-9. Although clinical studies have yet to show longitudinal data in chronically infected persons to support a direct role of immune function in HIV-1 control in vivo, much speculation has focused on the potential for increasing immune function in order to acquire better viral control and delay disease progression. Direct evidence for the role of cell-mediated immunity in the control of viral replication was obtained in the SIV animal model where depletion of CD8 T-cells resulted in a measurable increase of viral replication 10. In humans, a well-known cohort of patients in whom immune function is associated with viral control is in the subset of long-term non-progressors (LTNP) 11-14. However, confounding effects of the viral and genetic factors that may have contributed to their disease outcome make it difficult to conclude whether the levels of antiviral cellular immunity present in these individuals are the *reason for or consequence of* their viral control. This protocol addresses the potential role of immune function in controlling HIV-1 by testing the association between boosted antiviral immunity with control of viral replication in the absence of drugs. The long-term significance is to determine the safety of a novel approach to boost autologous antiviral responses in chronically infected persons (See Section B.1) and the clinical benefits of limiting drug exposure in patients if this strategy is found to be safe (See Section 2.1.a) whether it controls for viral replication or not. A short-term significance of the proposed pilot study is to determine whether boosted cell-mediated antiviral responses can be associated with viral control in the absence of therapy in chronically infected persons. Thus, whether or not the proposed hypothesis is correct, the results of this study will likely yield information relevant to immune-based therapies as well as intermittent/cyclic therapy strategies.

#### 2.2 HIV-1 Therapy and Immune-Mediated Control

The potential role of cellular immune responses to control HIV-1 replication has been proposed in light of data from studies of acute infection showing that viral-specific cytotoxic T-lymphocytes (CTL) are associated with a decrease in plasma viremia 15. High-level anti-HIV-specific immune responses are associated with a delay in the progression to AIDS in chronically infected individuals 16-19 as well as protection from infection in high-risk exposed individual 20,21. HIV-1 infected, “long-term non-progressors” (LTNP) maintain high CD4+ T cell proliferative responses to p24 antigen and to recall antigens, as well as high levels of HIV-1 tetramer, CD8 double positive T cells in association with a low viral loads (17; Ogg, personal communication). Unfortunately, the vast majority of HIV-1 infected individuals fail to suppress plasma virus below 40 copies/ml without therapy and lose their T cell proliferative responses soon after infection 22,23.

Anti-retroviral therapy has many beneficial effects in chronically infected persons. Irrespective of the disease stage during which it is started, anti-retroviral-mediated suppression can facilitate restoration of CD4+ T cell proliferative responses to recall antigens and can fill CD4 TCR Vβ repertoire gaps, among other effects 7,22,24-34. Recovery of immune function following HAART in chronically infected subjects has renewed the interest in augmenting anti-HIV-1 immune responsiveness in chronically infected persons. Increasing cell-mediated immunity against HIV-1 would be expected to delay disease progression and increase the efficacy of treatment by complementing anti-retroviral therapy-mediated suppression with immune-mediated control. Multiple strategies for boosting HIV-1 specific immune response under HAART are being explored 35-37. It remains undetermined how effectively these antiviral responses would be maintained in light of mounting data suggesting that prolonged HAART can result in a decline in HIV-1 specific cellular immune responses 38-40.

The association between cellular immune responses against HIV-1 antigens and temporary suppression of HIV-1 replication in the absence of therapy has been recently documented in acutely infected subjects who were treated within 120 days of infection and then had their treatment interrupted 41,42. Importantly, an association with viral replication and increased CD8-mediated cellular immune responses following temporary drug discontinuation was observed as reported by one of the co-investigators on this protocol 42. This observation, along with the observation of preserved and enhanced CD4^+^ T cell responses following initiation of suppressive therapy 22, has generated the hypothesis that periods of treatment interruption in acutely infected individuals may *preserve* and boost HIV-1 specific cellular immune responses in newly infected subjects. It has remained largely undetermined whether observations of boosted immune responses in acutely infected persons interrupting therapy are applicable to chronically infected persons in light of their longer duration of immune dysfunction, CD4+ T-cell loss, and viral replication. Taken together with Wistar preliminary data, this current study is significant to the application of this novel strategy for boosting antiviral immunity in chronically suppressed patients; Wistar preliminary data suggest that a subset of chronically infected persons can boost antiviral responses following therapy interruption yet its association with viral control is undetermined. It is also important to note that case reports on the association between multiple therapy interruption periods and immune-mediated control are predominantly focused on favorable outcomes 41,42, since no data are available on the frequency of side-effects on a group of individuals interrupting therapy in a similar manner. Since safety is a prime concern regarding any new therapy strategy, this protocol will significantly contribute to this area by intensely monitoring for the emergence of viral resistance, declines in CD4 count, and the clinical onset of disease progression events (i.e., opportunistic infections).

In addition to safety, this study’s primary aim is to measure time to rebound in viral load following sequential STIs. Secondary aims will target viral resistance, T cell activation and thymic function. Overall, observational data collected and reported by this group and others strongly justify the proposed objectives to expand the limited knowledge base on the safety, immune and viral consequences of structured treatment interruption as a novel treatment strategy in chronically suppressed individuals.

- 1. HIV-1 Therapy and Toxicity

Treatment guidelines for HIV-1 infection are centered on achieving viral suppression using highly active anti-retroviral therapy (HAART), based on the association between viral suppression and improved clinical outcome 43-48. Thus, the current goal of HAART is to sustain life-long suppression without treatment interruption with the hope of viral eradication if therapy is sustained. The feasibility of life-long HAART-mediated viral suppression is, however, limited by adverse drug effects, treatment cost, and the difficulty of maintaining optimal adherence for a prolonged duration 49,50. Moreover, the recent discovery of a long-lived latent reservoir of HIV-1 and a low level of viral replication in spite of undetectable plasma viremia has raised concern about the feasibility of viral eradication with the use of anti-retroviral regimens 2,51.

In addition to difficulties with adherence leading to drug resistance, use of antiretroviral drugs can result in unintended side effects. Short-term complications such as pancreatitis, peripheral neuropathy, and cytopenias related to nucleoside reverse transcriptase inhibitors, have been described for years. In the past two years, unexpected endocrine and metabolic complications associated with successful antiretroviral therapy have been reported 52-55. Different investigators have associated factors such as duration of therapy 53, type of therapy 54, degree of viral suppression 56, and even demographic characteristics 57-59 with the metabolic syndrome of peripheral lipodystrophy. The apparently increasing incidence of these metabolic complications, combined with a lack of understanding of their pathophysiology and clinical implications, leaves many clinicians and patients in a quandary about treatment. Many patients debate the benefits of continuation of life-prolonging HAART regimens because of these disturbing side effects and the uncertainty about other long-term sequelae. Interruptions in therapy have resulted in amelioration of some of the manifestations of lipodystrophy and improved well-being among patients within 2-4 weeks of therapy interruption. A greater understanding of the safety, viral and immune consequences of therapy interruption would aid clinical care of chronic HIV-1 infection by providing objective information to complement clinician/patient discussions of this practice when prompted by toxicity or quality of life considerations. Taken together, although many antiretroviral drugs with established virologic efficacy as part of combination regimens are now available, this study will address the urgent need to develop innovative ways to avoid the known and unknown limitations that make them largely impractical for maintaining long-term well-being in chronically infected subjects. That is, this protocol will be exploring a way of decreasing exposure to these clearly beneficial, but potentially harmful medications.

2.4 HIV-1 Therapy and Adherence Monitoring

Adherence to therapy is an important factor to consider in the interpretation of the effects of therapeutic interventions, such as structured therapy interruption. It is of interest to note that outcomes of increased antiviral immune response in early infection following periods of complete therapy interruption (See Section 2.1.a) lack any definitive measure of therapy adherence to rule out any contributing effects of poor adherence prior to, or in-between, periods of therapy interruption. However, assessment of pill-taking is a difficult problem in clinical medicine and research, because most methods have severe limitations. Several methods have been used in the past, either alone, or in combination with other techniques; no “gold standard” measure, however, currently exists for the study of most medications 60-64**.** Directly observed therapy is the closest of the methods to being a “gold standard,” yet, it is impractical for the majority of scenarios.

Each of the other following methods reviewed has limitations affecting its validity as a measure of dose-taking behavior. First, patient self-reporting is limited by the problems of both inaccurate recall and intentional deception. The latter is generally to avoid the stigma of having to admit to missing doses. While some poor adherers can be identified through non-judgmental questioning, doubts regarding the validity of self-reports persist due to the potential for over-reporting adherence 63. Second, pill counts are another commonly used technique. Unfortunately, a “correct count” does not rule out lost, discarded, or doubled doses. Some studies of pill-taking have found this method useful, while others have not 65-69. Both pill counts and patient self-reports have frequently been shown to over-report adherence, for a variety of reasons 70. Third, serum drug level monitoring is most relevant in studies looking at the emergence of resistant organisms since chronic low levels of drug are thought to predispose to the development of resistance; this phenomenon applies to HIV, and is analogous to that seen with tuberculosis treatment 71-75. Unfortunately, when studying medications with relatively short half-lives like most HIV drugs, measuring serum drug levels would yield information limited to the most recent doses.

In contrast, the advent of electronic monitoring systems has allowed an unbiased assessment of pill bottle opening and as such is one of the most valid surrogate markers of self-administered pill-taking 76,77. Equally importantly, the electronic measure allows for the evaluation of the pattern of pill ingestion, which has been shown in other diseases to have an important impact on outcome ^78,79^The Medication Event Monitoring System (MEMS -APREX Corporation, Menlo Park, CA) is a commercially available electronic recorder of the time and day of each opening, residing in the cap of a pill bottle (MEMS Track Cap). There are, of course, limitations to the MEMS Track Cap technology. This method assumes that each time the bottle is opened, a dose is taken, and that doses are not taken when the bottle is not opened. Thus, if an individual desires to “game” the system, adherence will be measured inaccurately. In addition, if the caps are lost or broken, the adherence data are lost. However, despite these limitations, several studies have established the validity and clinical significance of this measure 79-83.

## 3.0 RATIONALE

The working hypothesis of the proposed STI strategy is that a series of three STIs in chronically suppressed patients will result in increased antiviral immunity and better viral control is based on each STI acting as an independent boost of HIV-1 immunity as a result of the ensuing viral antigenemia and the immunological benefits of sustained suppressive therapy. Although Wistar preliminary data support the maintenance of boosted anti-HIV-1 cell mediated responses after a 46-day period of therapy interruption in patients with low anti-HIV-1 cell-mediated responses at baseline, controls indicate that the breadth and level of these responses may be lost in the absence of re-initiated therapy. As suggested in acute infection studies 22, therapy re-initiation may be a critical factor in preserving CD4 T-cell responses against HIV-1 p24 and gp-160. The rationale for the duration of each period of STI is based on Wistar observational data of the immunological outcome of time off therapy, the concept of prime and boost used in vaccination schedules, and the goal to restrict proposed periods without therapy.

The rationale for the initial two-week STI in the experimental group is not to allow viral rebound to achieve a high level or affect CD4 count in accordance with our preliminary data (safety STI). The rationale for establishing a uniform criterion to proceed with subsequent STI within five weeks of achieving viral suppression is to allow for the maximal amount of antiviral activated T-cells to be present at the time of the next viral re-bound. However, a variation in each individual’s time period to <50 copies/ml after an STI as determined by their individual response to anti-retroviral therapy and their immune-mediated set-point of viral control should be anticipated. The second STI period of 4 weeks is based on Wistar data showing that 28 days can be a sufficient period to boost pre-existing CD4 responses against p24 antigen. The third STI of 6 weeks is based on Wistar observational data showing 6 weeks off therapy can be maintained without a decline in CD4 while acting to significantly boost CD8 responses. In addition, Wistar observational data indicate that CD8 responses were boosted after a period greater than 28 days from treatment withdrawal.

The rationale for the definition of viral-rebound time as the first of three consecutive weekly viral loads above 5000 copies/ml is based on the lack of evidence that a period of three weeks above 5,000 copies/ml has any added hazard than those inherent to therapy interruption and the observational evidence by this group and others (J.M. Gatell and B. Walker, personal communications) that an initial rebound in individuals with prior treatment interruptions may decrease on its own in the subsequent two weeks. Thus, the latter definition of viral re-bound aims to not bias our analysis against detecting viral control if an acute viral load above 5,000 copies/ml can be associated with achieving subsequent suppression

.

The main rationale for follow-up of controls during a 40-week period before interruption in therapy is to increase confidence in the comparison between group outcomes by establishing viral suppression for an estimated period of 9 months. The latter would rule out at analysis participants who may be attracted to join the study due to their lack of adherence yet remain adequately suppressed at <50 copies/ml. An added benefit to follow-up of the control group is the equally timed control samples for the proposed secondary aims centered on comparison of viral and immune outcomes between groups.

Analysis will center on an intent to treat fashion of time to rebound and viral set-point. Based on Wistar preliminary data and that of others 84 it is anticipated that a viral load above 5,000 copies/ml may be achieved within two weeks of therapy interruption. Thus, making a four week determination of viral load possible since patients will be scheduled for weekly visits and the criteria for re-initiating therapy would not be met by the fourth week. Secondary analysis of this protocol will center on descriptive data on the viral and immune consequences of therapy interruption as measured by laboratory assays of antiviral immune response, indirect measures of thymic function, T-cell cell surface antigen levels, and viral genotyping before and during STI periods.

4.0 STUDY DESIGN & SAMPLE SIZE CALCULATION

This protocol is centered on addressing immunologic and virologic outcomes in a single center, randomized, non-blinded study comparing a strategy of sequential structured treatment interruptions (STIs) versus continuous viral suppression. Forty-two subjects with CD4 >400 cells/mm^3^ with a nadir CD4 ≥100 cells/mm^3^ and having maintained viral suppression while on a Highly Active Antiretroviral Therapy (HAART) (VL < 500 copies/ml for 6 months and <50 copies/ml at recruitment) will be randomized in a 1:1 fashion to either (1) three successive treatment interruptions of 2, 4, and 6 weeks, respectively, or (2) maintenance of HAART. Randomized distribution will help address the potential bias of including patients who have had prior treatment interruptions yet fulfill all entry criteria. The successive interruptions will be timed so that they will be sequentially instituted the week after two consecutive HIV RNA measurements of < 50 copies/ml, measured two weeks apart. The primary analysis will be an intent-to-treatment” analysis comparing the time to viral rebound (>5,000 for three consecutive weeks without 0.5 log decrease in viral load between samples) after a final period of a therapy interruption in both groups.

The sample size required was calculated using the Power program 85, and based on a type I error of 0.05, with a minimum of 80% power, to detect the smallest difference in time to rebound of viral load that would be considered clinically relevant (See Section C.1.1). Based on recent research 84 in persons interrupting therapy and our preliminary data, the time to viral rebound in the control group is expected to be on the order of 2 weeks. Table below depicts the required sample size per study group for a range of detectable differences for either 80% or 90% power. Thus, 13 subjects per group, will afford us more than sufficient power to determine if a difference of 1 month or greater exists between the experimental and control groups in time to rebound of virus during the open-ended 4^th^ therapy interruption in the experimentals and the 1^st^ interruption period in controls. Assuming a conservative drop-out rate of 15%, we have increased the planned enrollment to 21 per group, or 42 total.

Sample size calculation

| Detectable Difference  Between Groups | 80% Power | 90% Power |
| --- | --- | --- |
| 4 weeks | 13 | 18 |
| 5 weeks | 10 | 14 |
| 6 weeks | 8 | 11 |

Although safety will remain a primary focus of the analysis, the study is powered for the virological outcome rather than a safety outcome due to Wistar preliminary safety data from monitoring periods without therapy that exceeded those used in this protocol. Safety will be assessed by changes in CD4 count, degree of viral rebound in response to treatment cessation, and symptomatic adverse events and will be monitored by a Data and Safety Monitoring Board (DSMB). Immunological outcomes will be assessed by measurement of antiviral CD4 and CD8 responses, cell surface antigen measurements of T-cell subsets and indirect measures of thymic output. Virological outcomes will be measured by viral load and viral genotyping at each rebound during STI. Overall, the proposed analysis is primarily centered on determining the safety, immunologic and virologic consequences of treatment interruption as a first step towards evaluating the role of this intervention in clinical management of chronic HIV-1 infection.

# 5.0 SELECTION AND ENROLLMENT OF SUBJECTS

## 5.1 Inclusion Criteria

5.1.1 Age greater than 17 years.

5.1.2 Able and willing to provide informed consent.

5.1.3 Documented HIV-1 infection by at least two different licensed tests, including an ELISA, and confirmed by Western Blot, HIV-1 culture, HIV-1 antigen, plasma HIV-1 RNA or HIV-1 DNA by PCR.

5.1.4 HIV RNA < 500 copies/ml on a regimen of 2 Nucleoside Reserve Transcriptase Inhibitors (NRTIs) and either 1 PI or 1 Non-NRTI (NNRTI) for 8 months.

5.1.5 HIV RNA < 50 copies/ml at screening.

5.1.6 >400 CD4 with CD4 nadir of > 100 cells within the last 36 months.

5.1.7 Documentation of previous HIV-1 VL >10,000 copies/ml at any time prior to initiating the current uninterrupted HAART regimen.

5.1.8 Both male and female subjects are eligible. All female subjects enrolled must have a negative pregnancy test (β-hCG) within 2 weeks of therapy interruption AND must agree to use appropriate contraceptive methods while on study. All female subjects will be informed of potential risks to the developing fetus incurred both when off therapy, and when on combination therapy.

5.1.9 Able to carry out “normal activities of daily living” with minimal effort as assessed by intake physician (Karnofsky performance scale score of 80% or better).

5.1.10 Subjects must be willing to adhere to the treatment and treatment interruption schedule and HAART re-initiation regimen and schedule approved by the study investigators in conjunction with the patient’s primary provider. The latter confirmation of willingness will not impact the patients’ right to withdraw from the study at any point.

5.1.11 Subjects must be willing to abstain from all immunomodulatory drugs (defined below) during the study period

## 5.2 Exclusion Criteria

5.2.1 Currently pregnant or breast feeding.

5.2.2 Previous CD4 nadir < 100 cells/mm^3^ .

5.2.3 History of immunomodulatory therapy within the last 4 weeks, including, but not limited to: systemic corticosteroids; systemic cancer chemotherapy/irradiation; cyclosporin; tacrolimus (FK-506); OKT-3; any Interleukin, including IL-2; any Interferon; cytoxan (cyclophosphamide); methotrexate; IVIG (gamma globulin); G/M-CSF; hydroxyurea; thalidomide; pentoxifylline; thymopentin; thymosin; dithiocarbonate; polyribonucloside.

5.2.4 Significant co-existing medical condition, including:

- - - Anemia (Hgb <9.1 men, <8.9 women)
    - Neutropenia (ANC < 1000)
    - Thrombocytopenia (platelet count <50K)
    - AST/ALT > 5 x ULN
    - Total Bilirubin > 1.5 UL
    - Total Bilirubin >3 x ULN if receiving indinavir
    - Renal disease (creatinine > 2x upper normal limits)
    - Other conditions, such as active drug/alcohol abuse or dependence which, in the opinion of the investigator, would interfere with study compliance.

## 5.3 Study Enrollment Procedures

5.3.1 Prior to implementation of this protocol, the protocol and consent form must be approved by the Philadelphia FIGHT Institutional Review Board (IRB).

- - 1. Once a candidate for study entry has been identified, details will be carefully discussed with the subject by a scheduled visit with the Research Nurse to be followed by a visit with a clinician on the study. The subject will be asked to read and sign the consent form that was approved by the Philadelphia FIGHT IRB. The subject will be provided with a copy of the Informed Consent. Informed consent will be obtained prior to the performance of any study-related procedures.
    2. Patients will be recruited through Philadelphia FIGHT utilizing a network of clinics in Philadelphia. The patient contact site will be Philadelphia FIGHT’s Jonathan Lax Immune Disorders Treatment Center which has more than four years of experience conducting clinical research (86) in a patient community educated on the value of clinical research. The patient community at this site is composed of 41% females, 62% African Americans, and 8% Hispanics with a predominance of 4-8 years of diagnosed HIV-1 infection and 25-45 years of age.

# 6.0 STUDY TREATMENT

## 6.1 Medication Requirements

- - 1. The protocol requires stable highly active antiretroviral therapy (HAART) for a period of 6 months prior to study entry. There are no experimental drugs required with this study. All the medications in the various HAART regimens have been approved by the Food & Drug Administration (FDA). The cost of HAART medications will not be provided by this study. Subjects will receive instruction on the major classes of antiretroviral medications and their toxicities in the informed consent. The information provided will include the following information.
       1. Nucleoside Reverse Transcriptase Inhibitors (NRTIs)

This class of drugs can result in abnormal levels of acid in your blood (lactic acidosis) and affect liver function (severe hepatomegaly or enlarged liver) increasing the amount of fat in the liver (steatosis or fatty liver) that may result in liver failure. Other complications and death have been reported with the use of nucleoside reverse transcriptase inhibitors (NRTIs) alone or in combination. This class of anti-HIV drugs includes ZDV (AZT, Zidovudine or Retrivir™), ddI (Didanosine or Videx™), ddC (Zalcitabine, or Hivid™), 3TC (Lamivudine or Epivir™), d4T (Stavudine or Zerit™) and ABC (Abacavir or Ziagen™).

6.1.1.2 Non-Nucleoside Reverse Transcriptase Inhibitors (NNRTIs)

Another class of drugs which inhibits reverse transcriptase in the life cycle of HIV is commonly referred to as the “non-nukes”. Many patients experience a mild rash upon starting therapy. Other side effects include fever, muscle soreness, body ache, headache, light-headedness, insomnia, abnormal dreams, nausea, diarrhea and elevated liver function. In very rare cases, a potentially life-threatening skin condition known as Stevens-Johnson syndrome has been reported. This class of anti-HIV drugs includes NVP (Nevirapine or Viramune™), DLV (Delavirdine or Rescriptor™) and EFV (DMP-266, Efavirenz or Sustiva™).

- - - 1. Protease Inhibitors (PIs)

The use of potent antiretroviral drug combinations, such as HAART, usually includes a protease inhibitor. This class of anti-HIV drugs includes AMP (Amprenavir or Agenerase™), IDV (Indinavir or Crixivan™), NFV (Nelfinavir or Viracept™), RTV (Ritonavir or Norvir™), and SQV (Saquinavir or Fortovase™). Protease inhibitors (PIs) have been associated with an abnormal distribution (placement) of body fat and wasting (thin appearance). Some of these body changes include an increase in fat around the waist and stomach area, increase in fat on the back of the neck, breast enlargement and thinning of the face, legs and arms. The use of HAART regimens that commonly contain a PI has also been associated with changes in fat metabolism including elevated triglycerides (increased fatty acid in the blood) and/or elevated cholesterol. The use of protease inhibitors may be associated with the development or worsening of elevations in blood sugar and diabetes. There have been reports of increased bleeding in HIV-infected persons with bleeding disorders (hemophilia) who were treated with PIs. It is not known if PIs were the cause of these bleeding episodes.

- - 1. Dosing adjustments

If the subject experiences any side effects or other symptoms between scheduled study visits, the subject will be instructed to report them immediately to the research physician or research nurse. In collaboration with the subject’s primary care provider, the investigator may decide to adjust the dose or dosing schedule of the subject’s HAART medications. The subject will be informed that the side effects listed below are the most common or most serious seen with the different drug classes. The subject will be instructed to contact the study nurse or study doctor concerning any questions about any additional side effects the subject may experience during participation in this study.

6.1.3 Prohibited Medications

History of immunomodulatory therapy within 4 weeks prior to study entry and for the duration of participation in the protocol including, but not limited to: systemic corticosteroids; systemic cancer chemotherapy/irradiation; cyclosporin; tacrolimus (FK-506); OKT-3; any Interleukin, including IL-2; any Interferon; cytoxan (cyclophosphamide); methotrexate; IVIG (gamma globulin); G/M-CSF; hydroxyurea; thalidomide; pentoxifylline; thymopentin; thymosin; dithiocarbonate; polyribonucloside.

6.2 Clinical Modulation of Treatment

6.2.1 Control Arm

After randomization, those individuals randomized to continuation of HAART will be followed every 4 weeks for 40 weeks. HIV-1 viral load, CBC, CD4 count test schedule as well as biological sample collection are listed in Table 1. During the study period, data on adherence to antiretroviral therapy will be obtained every four weeks through the use of the Medication Event Monitoring System (MEMS -APREX Corporation, Menlo Park, CA), and at each visit data on any changes in clinical status will be obtained. Any changes in antiretroviral therapy will be recorded as well. If during this 40-week period, HIV RNA rebounds to >1,000 measured two weeks apart or greater than 50 copies/ml for a period exceeding 6 weeks the subject will be removed from the study. Within a week from the 40th week visit, patients who remain with a viral load less than 50 copies/ml will have therapy withdrawn (NNRTIs are to be stopped 24 hour prior that the rest of medications). During this comparison treatment interruption (CTI), patients will be monitored at weekly visits to assess for any clinical changes. Weekly samples will be drawn for CD4 cell counts, HIV RNA and CBC until HIV RNA is > 5,000 copies/ml without evidence of a decrease of 0.5 log between measurements within a three week period. Reinitiation of therapy will occur when viral load was >30,000 copies/ml for three consecutive time points. Following re-initiated therapy, follow-up on therapy to confirm resuppression to <50 copies/ml at 6, 10 and 14 weeks on therapy will be performed. If less than 0.5 log difference from peak viral load is observed at week 6 of reinitiated therapy, GART testing will be offered.

6.2.2 Experimental Arm

Those individuals randomized to the sequential STI group will have three sequential STIs in the following fashion (note NNRTIs are to be stopped 24 hour prior that the rest of medications):

- - - 1. Priming/Safety STI-

Within 4 weeks of randomization and acquisition of baseline samples, therapy will be interrupted for 2 weeks. Following 14 days without therapy, therapy will be re-initiated until a week after 2 successive HIV RNA measurements < 50 copies/ml measured two weeks apart. Subjects who do not show greater than 0.5 log reduction in viral load by 6 weeks of re-initiated therapy or who do not achieve <50 copies/ml by 20 weeks of therapy will be offered GART testing. Subjects who do not achieve <50 copies/ml by 20 weeks will be withdrawn from the study.

Within a week of 2 successive HIV RNA tests at < 50 copies/ml as a result of re-initiated therapy following the priming STI period, therapy will be withdrawn for 4 weeks. After therapy re-initiation, therapy will be continued until HIV RNA is < 50 copies/ml for 2 successive two-week intervals. Subjects who do not show greater than 0.5 log reduction in viral load by 6 weeks of re-initiated therapy or who do not achieve <50 copies/ml by 20 weeks of therapy will be offered GART testing. Subjects who do not achieve <50 copies/ml by 20 weeks will be withdrawn from the study.

- - - 1. CD8 boost STI-

Within a week of 2 successive HIV RNA tests at < 50 copies/ml as a result of re-initiated therapy following the CD4 boost STI period, therapy will be withdrawn for 6 weeks. After therapy initiation, therapy will be continued until HIV RNA is < 50 copies/ml for 2 successive two-week intervals. Subjects who do not show greater than 0.5 log reduction in viral load by 6 weeks of re-initiated therapy or who do not achieve <50 copies/ml by 20 weeks of therapy will be offered GART testing. Subjects who do not achieve <50 copies/ml by 20 weeks will be withdrawn from the study.

- - - 1. Comparison treatment interruption –

Within a week of 2 successive HIV RNA tests at < 50 copies/ml as a result of re-initiated therapy following the CD8 boost STI period, therapy will be withdrawn and patients will be monitored weekly for up to 10 weeks to assess clinical changes. Weekly determinations for CD4 cell counts, HIV RNA and CBC will be monitored until HIV RNA is > 5,000 copies/ml without evidence of a decrease of 0.5 log between measurements within a three week period. The first week in the series will be defined as the time to rebound in this group. Reinitiation of therapy will occur when viral load was >30,000 copies/ml for three consecutive time points.

- - - 1. Six-Week Follow-Up –

Following re-initiated therapy, follow-up on therapy to confirm resuppression to <50 copies/ml at 6, 10 and 14 weeks on therapy will be performed. If less than 0.5 log difference from peak viral load is observed at week 6 of reinitiated therapy, GART testing will be offered.

7.0 CLINICAL EVALUATION AND LABORATORY EVALUATIONS

A symptom-directed history and physical exam will be documented at the time of entry. These will be repeated at Day 0 and then monthly. Past history including HIV-related and non-HIV-related diagnoses, prior anti-HIV therapies, immunomodulatory therapies and vaccines, current prescription medications and laboratory reports concerning year of diagnosis, viral loads and CD4 counts will be obtained. Medical records will be reviewed to determine peak HIV-1 viral load and nadir CD4 count. History of opportunistic infections will also be obtained from the records.

7.1 Control Arm

**Table 1.**  Listing of visit requirements and case report forms used for the control group assuming a viral rebound not to exceed 10 weeks of treatment interruption during the comparison treatment interruption period.

7.2 Experimental Arm

**Table 2.**  Listing of visit requirements and case report forms used for the experimental group assuming a viral rebound not to exceed 10 weeks of treatment interruption during the comparison treatment interruption period.

Table 2. (Continued)

7.3 Data Collection and Biological Samples

Clinical data regarding viral load, CD4 count and clinical events will be collected every two weeks from entry at regularly scheduled visits to Philadelphia FIGHT as previously summarized in Table 1 and Table 2 .

Biological sample collection for laboratory analysis of immune and viral outcomes will be derived from a total of eight 10ml heparinized venous peripheral blood samples (independent of blood draws for viral load, CBC and CD4 count) which yield 8 mls of blood per tube for an anticipated yield of 70-80x10^6^ PBMC in addition to plasma serum. Blood volume collected will be used as 1 ml of whole blood for flow cytometry and 15x10^6^ PBMC for lymphoproliferative recall responses on the day of collection. The remainder PBMC yield will be stored as two cryopreserved 5x10^6^ PBMC aliquots and two 20x10^6^ PBMC that will be used for measures of antiviral CD8 activity, indirect measures of thymic function and quantitation of viral reservoirs at designated time-points. Plasma will be stored as four 2ml aliquots for viral genotype studies and for the availability to repeat clinical tests in the event of loss in shipment.

7.4 Frequency of Sampling and Amount of Blood That Will Be Drawn

The frequency of sampling was determined necessary by the inability to anticipate the responses of each individual following treatment interruption, the need to minimize the risks to patients by close monitoring, and the need to identify the time to undetectable viral loads after re-initiation of therapy in order to proceed to the next STI period. Table 3 identifies the anticipated frequency and amount of blood that will be obtained in the control group.

The individualized progression through the protocol in the experimental arm as a consequence of sequence of events as defined by viral load results in a variable times & number of blood draws for each subject. We provide a minimum (Table 4) and a maximum (Table 5) listing of blood draws that can take place according to the protocol.

The proposed frequency of sampling is based on the safety of participants, and the protocol needs for blood to be drawn were approved by the Philadelphia FIGHT IRB and the Wistar Institute IRB. The amount of blood drawn in the protocol was estimated using the following criteria:

7.4.1 A reduced amount of blood will be collected during re-initiation of therapy periods if the previous viral load was higher than 50 copies/ml within 4-week intervals. This will reduce the blood drawn to 64 ml every other scheduled visit if the patient is on therapy yet has not met criteria to proceed to the next interruption period.

7.4.2 A reduced amount of blood will be collected during weekly samplings in the comparison interruption period if the previous viral load was lower than 5,000 copies/ml within 2- week intervals. This will reduce blood draws to 64 ml every 4 weeks if the patient is not on therapy during the comparison interruption period and has not met criteria to re-initiate treatment.

- - 1. Overall, these changes will significantly reduce the total blood collected for both the control and experimental groups over the duration of the study from that which was originally proposed. Any further reductions in blood draws would compromise safety and the scientific objectives of the protocol in regards to investigating immune and viral outcomes of therapy interruption.

**Table 3.** Blood Draw Summary for Control Arm

Listing of biological sample collection in control group. Example of sample distribution for control group over a projected 61-week time period assuming a viral rebound by 2-4 weeks of treatment interruption during the comparison treatment interruption period.

Stage Week LabCorp Wistar

Baseline (-4) on Tx. -4 17ml 64ml

Baseline (-2) on Tx. -2 17ml 64ml

Monitor 0 17ml 64ml

Monitor 4 17ml 64ml

Monitor 8 17ml 64ml

Monitor 12 17ml 64ml

Monitor 16 17ml 64ml

Monitor 20 17ml 64ml

Monitor 24 17ml 64ml

Monitor 28 17ml 64ml

Monitor 32 17ml 64ml

Monitor 36 17ml 64ml

Monitor 40 17ml 64ml

Comparison-STI 41 17ml 64ml

Comparison-STI 42 17ml

Comparison-STI 43 17ml 64ml

Comparison-STI 44 17ml

Comparison-STI 45 17ml 64ml

Comparison-STI 46 17ml

Comparison-STI 47 17ml 64ml

Comparison-STI 48 17ml

Comparison-STI 49 17ml 64ml

Comparison-STI 50 17ml

Comparison-STI 51 17ml 64ml

Viral Load at 6 wks of Re-initiated therapy 5ml

**TOTAL ML 413ml 1,216ml**

**TOTAL BLOOD DRAW OVER 61 WEEKS: 1,629 ml**

**Table 4** Minimum Blood Draw (46 week protocol) -- Example of sample distribution for experimental group over a projected 42-week time period assuming<50 copies/ml HIV-1 RNA is achieved by 4 weeks (minimum) of reinitiated therapy following each STI.

# Stage Week LabCorp Wistar

Baseline (-4) on Tx. -4 17ml 64ml

Baseline (-2) on Tx. -2 17ml 64ml

Start Prime-STI(0) 0 17ml 64ml

Re-Start Tx. 2 17ml

Tx 4 17ml 64ml

Tx 6 17ml 64ml

CD4-STI 7 17ml 64ml

CD4-STI 9 17ml 64ml

Re-Start Tx. 11 17ml

Tx 13 17ml 64ml

Tx 15 17ml 64ml

CD8-STI 16 17ml 64ml

CD8-STI 18 17ml 64ml

CD8-STI 20 17ml 64ml

Re-Start Tx. 22 17ml

Tx 23 17ml 64ml

Tx 25 17ml 64ml

Comparison-STI 26 17ml 64ml

Comparison-STI 27 17ml

Comparison-STI 28 17ml 64ml

Comparison-STI 29 17ml

Comparison-STI 30 17ml 64ml

Comparison-STI 31 17ml

Comparison-STI 32 17ml 64ml

Comparison-STI 33 17ml

Comparison-STI 34 17ml 64ml

Comparison-STI 35 17ml

Comparison-STI 36 17ml 64ml

Viral Load at 6 wks of Re-initiated therapy 5ml

TOTAL ML (52 weeks) 481ml 1,280ml

**TOTAL BLOOD DRAW OVER 46 WEEKS: 1,761 ml**

**Table 5.** Maximum Blood Draw (89 week protocol) -- Example of sample distribution for experimental group over a projected 89-week time period assuming<50 copies/ml HIV-1 RNA is achieved by 20 weeks (maximum) of reinitiated therapy following each STI.

Stage Week LabCorp Wistar

Baseline (-4) on Tx. -4 17ml 64ml

Baseline (-2) on Tx. -2 17ml 64ml

Start Prime-STI(0) 0 17ml 64ml

Re-Start Tx. 2 17ml

Tx 3 17ml 64ml

Tx 5 17ml

Tx 7 17ml 64ml

Tx 9 17ml

Tx 11 17ml 64ml

Tx 13 17ml

Tx 15 17ml 64ml

Tx 17 17ml

Tx 19 17ml 64ml

Tx 21 17ml

CD4-STI 22 17ml 64ml

CD4-STI 24 17ml 64ml

Re-Start Tx. 26 17ml

Tx 27 17ml 64ml

Tx 29 17ml

Tx 31 17ml 64ml

Tx 33 17ml

Tx 35 17ml 64ml

Tx 37 17ml

Tx 39 17ml 64ml

Tx 41 17ml

Tx 43 17ml 64ml

Tx 45 17ml

CD8-STI 46 17ml 64ml

CD8-STI 48 17ml 64ml

CD8-STI 50 17ml 64ml

Re-Start Tx. 38 17ml

Tx 40 17ml 64ml

Tx 42 17ml

Tx 44 17ml 64ml

Tx 46 17ml

Tx 48 17ml 64ml

Tx 50 17ml

Tx 52 17ml 64ml

Tx 54 17ml

*(Continuation of table 5)*

Stage Week LabCorp Wistar

Tx 56 17ml 64ml

Tx 58 17ml

Comparison-STI 59 17ml 64ml

Comparison-STI 61 17ml

Comparison-STI 63 17ml 64ml

Comparison-STI 65 17ml

Comparison-STI 67 17ml 64ml

Comparison-STI 69 17ml

Comparison-STI 71 17ml 64ml

Comparison-STI 73 17ml

Comparison-STI 75 17ml 64ml

Comparison-STI 77 17ml

## Comparison-STI 79 17ml 64ml

## Viral Load at 6 wks of Re-initiated therapy 5ml

TOTAL ML (89 weeks) 889ml 1856ml

**TOTAL BLOOD DRAW OVER 89 WEEKS: 2,745 ml**

7.5 Study Discontinuation Procedures

Patients who discontinue from the study for any reason will be required to return to the clinic for a final evaluation. As indicated in Table 1 and Table 2, all assessments and specimen collections indicated at F/U x 6 (6 weeks after final re-initiation of HAART therapy) will be performed at that time.

# 8.0 CONCOMITANT_THERAPY

All prescription therapies and immunizations, as well as any other HIV-directed medications or treatments taken by the patient or administered to the patient during the study will be reported as concomitant treatment in study records. Over-the-counter medications will not be recorded unless an investigator feels that such medications or dietary supplements will provide significant information to assist in the analysis of the data.

# 9.0 ADVERSE_EVENTS_AND _MANAGEMENT

- 1. Adverse Events

An adverse event (AE) is defined as any untoward medical occurrence in a subject participating in a clinical study. It does not necessarily have to have a causal relationship with this treatment. An AE can, therefore, be any unfavorable and unintended sign (including an abnormal laboratory finding, for example), symptom, or disease temporally associated with the use of a medicinal product, whether or not considered related to the medicinal product. This definition includes intercurrent illnesses or injuries and exacerbations of pre-existing conditions. All grades of AEs, dose-limiting AEs, and serious adverse events (SAEs) must be recorded on the study case report forms (CRFs). All SAEs should be reported following the signing of the Informed Consent. Grades of severity will be recorded according to the modified WHO Common Toxicity Criteria (refer to Appendix 5).

An out-of-range laboratory value will be considered clinically significant if intervention is required, medications are prescribed, or a special course of action is taken for follow-up. A laboratory value with a toxicity of Grade 3 or 4 is usually considered clinically significant. If considered clinically significant, this abnormal laboratory result/diagnosis should be recorded as an adverse event.

9.1.1 Not Related:

The AE is not related if the occurrence of the AE is not reasonably related in time to a study-related interruption of or re-initiation of HAART therapy.

9.1.2 Possibly Related:

The AE is possibly related if the occurrence of the AE is reasonably related in time to a study-related interruption of or re-initiation of HAART therapy.

- - 1. Probably Related:

The AE is probably related if the occurrence of the AE is reasonably related in time and is more likely than other causes to be responsible for the AE, or is the most likely cause of the AE.in relationship to a study-related interruption of or re-initiation of HAART therapy.

9.2 Serious Adverse Event

All SAEs must be reported immediately (10 working days) to the Data Safety and Monitoring Board (DSMB) and IRB. In case of death of a study subject, a copy of the autopsy report should be supplied, if available. A serious adverse event (SAE) is defined as any untoward medical occurrence that:

- Results in death
- Is life threatening (ie, the subject was, in the opinion of the Investigator, at immediate risk of death from the event as it occurred)
- Requires or prolongs inpatient hospitalization
- Results in persistent or significant disability/incapacity (ie, the event causes a substantial disruption of a person’s ability to conduct normal life functions)
- Is an important and significant medical event that, based upon appropriate medical judgment, may jeopardize the patient.

9.3 Pregnancy

Recruitment excludes pregnancy from the protocol. However, pregnancies are not considered to be AEs or SAEs; however, pregnancies will be followed through outcome and applicable CRF pages must be completed. In pregnancies that progress to term, any congenital abnormalities in the offspring of a subject who participated in the study should be reported as an SAE.

- 1. Study Discontinuation

Subjects withdrawn from the study due to an AE or SAE will be followed by the investigator at least until a final outcome is determined (ie, sequelae are considered permanent) and reported to the DSMB and IRB and, if possible, until the study is completed.

# 10.0 CASE REPORT FORMS (CRFs)

10.1 Source Documents

Subject source documents are the physician’s patient records. CRFs will be provided for each subject. Subject data will be transcribed onto the CRFs in a timely fashion, and the study monitor will audit these records.

10.2 Review of CRF Pages

It is the Principal Clinical Investigator’s, or designated physician Sub-Investigator’s responsibility to review and approve all completed CRFs. The Investigator has the obligation to sign and date the CRF review page in each subject’s CRF book, verifying that the information is true and correct.

- 1. Changes in CRF Pages

Changes and/or additions to data entered on the original CRFs must be made in the following manner: The original entry will be struck out with a single line through the error (neither erasures nor correction fluid should be used) so as to leave it legible. The correction will be entered using a black ballpoint pen, initialed, and dated by the person making the correction. The Principle Clinical Investigator or delegate, such as Sub-Investigator or Study Coordinator may enter corrections on the original CRFs. If changes are made after review and signature by the Principal Clinical Investigator, the Principal Clinical Investigator must be made aware of the changes and document this awareness by re-signing and dating the CRF page.

- 1. Data Verification

The standard for this study will be that 100% of the study data must be verifiable with the source data, which necessitates access to all original recordings, laboratory reports and subject records. The investigators therefore agree to allow direct or indirect access to patient records, and source data must be made available for all study data, which will be considered confidential and which will be dealt with as such. The subjects must also allow access to their medical records and will be informed of this, confirming their agreement when signing the informed consent.

- 1. CRF Completion

All fields on the CRFs must be completed per the instructions provided on the CRF pages. The following abbreviations will be used when values or answers cannot be provided: NA or N/A (Not Available and Not Applicable), ND (Not Done), and UNK (Unknown).

# DATA COLLECTION

Data collection for clinical history, viral load, CBC and CD4 and CD8 count changes will be collected by the research nurse and recorded on case report forms that will be forwarded to the principal investigator.

12.0 SUBSTUDY DATA COLECTION

Based on the central role of anti-HIV-1 cell-mediated responses in the hypothesized biological effect of the STI strategy, measures of antiviral CD4 and CD8 T-cell responses will be performed at all contact times in both control and experimental groups. Tables 2 and 3 show the anticipated tests to be performed over the five-year period of the study. Our experience in monitoring anti-viral cell-mediated activity at each contact time, as presented in the preliminary data section, has allowed for a higher level of confidence in interpreting the effects of viremia and reinitiated therapy on antiviral cell-mediated immunity.

12.1 Data Collection for Recall Lymphoproliferative T-cell Responses (Dr. Montaner’s Laboratory)

In accordance with optimal conditions for lymphoproliferative response analysis 87, PBMC will be isolated and used the same day of blood collection. PBMC will be isolated by standard Ficoll-hypaque density gradient centrifugation, washed 3 times in sterile 1xPBS, resuspended in RPMI 1640 supplemented with 10% FBS, 100U/ml penicillin/100μg/ml streptomycin and 2mM glutamine, and cultured in 96 Falcon U-well plates at 250,000 cells/well. Each sample will be divided into nine sixtuplet groups of wells to include one unstimulated control and eight stimulated conditions as follows: seven groups stimulated with Candida, insect cell/baculovirus recombinant HIV-1 antigens [HIV-1 p24 core protein including additional amino acids of the C-terminus of p17 and N-terminus of p15 with molecular weight of 35,000 on SDS/PAGE (Protein Sciences), HIV-1 gp-160 LAV fully glycosylated with molecular weight of 160,000 on SDS/PAGE (Protein Sciences)], one positive control [PHA, phytoheamoglutinin (5μg/ml, Sigma)], and one negative control [protein control provided by the manufacturers of recombinant viral antigens (5 μg/ml, Protein Sciences, Meriden, CT)]. HIV-1 specific stimulations will be tested at 0.1, 0.5, 5 μg/ml. After 5 days in culture, all groups will be pulsed for 18hrs with tritiated thymidine (1 Ci/well). Nuclei harvested by lysis of PBMCs in each well in 70 μl of lysis buffer (0.5 M NaCl, 0.1% SDS, 10 mM EDTA, 1% Tween 20), and collection onto fiberglass filter paper (Packard, Meriden, CT) using an automatic multi-well harvester. Radioactivity in the filter paper will be quantified as counts per minute (c.p.m.) using a Packard Direct Beta Counter 9600, . Results will be expressed as stimulation index (SI=Antigen stimulated mean c.p.m./unstimulated mean c.p.m.) with an SI>3 considered positive and as delta c.p.m. (mean c.p.m. antigen stimulated – mean c.p.m. unstimulated control). Dr. Montaner’s laboratory has over 4 year experience with these methods and analysis. Anticipated problems center on the limitations of interpreting frequencies of CD4 T-cell responses by this method and the potential for biasing against higher levels of response by the 5-day culture period required before data can be collected. The first problems have been addressed by testing three decreasing concentrations of antigen and performing intracellular cytokine recall assays by use of flow cytometry. Although the intracellular cytokine assay for recall responses can provide more quantitative characterization of CD4 T-cell responses against HIV-1 antigens, investigators elected not to use this test in the primary analysis of CD4 responses due to: (1) a greater variability in this assay’s performance for HIV-1 antigen stimulations over the last 10 months of experience using it in parallel with lymphoproliferative assays, and (2) extensive use and characterization of the lymphoproliferative test in patients interrupting therapy as the basis for our STI strategy. The latter is the main justification for choosing lymphoproliferation as the primary assay of antiviral CD4 responses.

- 1. Data Collection for Antiviral CD8 T-cell Responses (Dr. Nixon’s Laboratory)

Anti-HIV-1 specific cytotoxic T cell (CTL) activity will be quantified by using ^51^chromium release assays, staining of PBMC with MHC tetrameric peptide complexes, and ELISPOT to measure release of Interferon-gamma. An alternative method to quantify virus-specific T cells is to detect intracellular cytokines released after specific antigen stimulation by flow cytometry 88,89. All patients will have the ELISPOT assay and staining of PBMC with tetramers (for HLA haplotypes for which tetramers are available). ^51^Chromium release assays will be performed in selected subjects where a successful EBV transformed B cell line has been established. Samples will be collected and processed the same day at the Wistar Institute; PBMC will be cryopreserved and shipped to ADARC for CTL analysis.

12.2.1 Cell lines & ^51^Chromium Release Assays.

Autologous B-lymphoblastoid cell lines (BCL) from study subjects are transformed by incubating peripheral blood mononuclear cells (PBMC) with RPMI 1640 containing 15% fetal bovine serum (FBS), 10mM Hepes, 2mM L-glutamine and 50IU of Pen/Strep, and Epstein-Barr virus from the supernatant of the B95/8 cell line. ^51^Chromium release assays will be performed as previously described 42,89

12.2.2 Tetrameric peptide complex staining38,39,90,91.

A set of tetramers with HIV peptides restricted by the following HLA alleles: HLA-A2, A3, A11, B7, B8, B27 will be used. PBMC are analyzed for the expression of cell surface markers and tetramer-positive cells using a Fluorescence Activated Cell Sorter (FACS) Calibur (Becton Dickinson) with CellQuest software (Becton Dickinson). Tetramers are provided by Dr. Ogg, University of Oxford. Staining of PBMC identifies a discrete population of CD8/tetramer double positive cells. The lower limit of detection of antigen-specific CTL using the HLA-tetramers is approximately 0.02% of CD8+ cells, based on the staining of HIV-1-negative PBMC 38. Gates are applied to contain >99.98% of the negative controls (HLA mismatched PBMC) . HIV specific CTL clones or lines of known specificity are used as positive controls for tetramer stains.

12.2.3 ELISPOT assay.

Anti-HIV-1 specific CD8 T-cell activity will be quantified by using enzyme-linked immunoassay (ELISPOT) to measure release of Interferon-gamma as described 42,89. Briefly, PBMC (10^7^/ml) are incubated for 1 hour at 37^0^C in RPMI 1640 + 1% pooled human serum (PHS) with rVV expressing HIV-1 antigens, at a multiplicity of infection (MOI) of 2. Uninfected and infected PBMC are added to the anti-IFN-γ-coated wells in 100-200 μl culture medium and incubated overnight at 37^0^C in 5% CO_2_. Wells are washed, incubated with secondary antibody (1mg/ml, Biotin conjugated anti-IFN-γ mab; Mabtech, Stockholm, Sweden), washed, developed and counted with a stereo microscope. Only spots with a fuzzy border and a brown color are counted. IFN-γ producing cells detected in this assay are predominantly CD8+ T cells 89. Results are expressed as spot forming cells (SFC) per 10 ^6^ PBMC.

12.2.4 Cytokine flow cytometry 92,93.

For CD8+ functional studies, PBMC are stimulated with HIV-1 specific CTL epitope peptide at a concentration of 10 μg/ml in the presence of antibody to CD28. The incubation is done at 37^o^C in 5% CO_2_ with the last five hours performed in the presence of Brefeldin A (Sigma) at a final concentration of 10μg/ml. Following stimulation, cells are washed in PBS, incubated for five minutes at 37^o^C in 0.02% EDTA and washed in PBS. Cells are then fixed briefly in 1% paraformaldehyde (PFA), washed in 1% BSA, and frozen for at least 18 hours in 1% BSA containing 10% DMSO ). Cells are then thawed, washed with 1% BSA, sequentially incubated for 10 minutes in FACSLyse and FACSPerm solutions (both Becton-Dickinson), and washed in 1% BSA. Finally, cells are stained with antibodies to CD4 (conjugated to fluoroscein isothiocyanate:FITC or allophycoerythrin:APC), CD8 (conjugated with FITC or APC), CD69 (conjugated with peridinin chlorophyll protein: PerCP), and TNF (conjugated to phycoerythrin:PE) (all antibodies are obtained from Becton-Dickinson except CD4-APC from Exalpha, Boston, MA), fixed in 1% PFA, and collected on a FACSCalibur flow cytometry instrument using CellQuest software (both Becton-Dickinson), as previously described 28. Flow cytometry data is analyzed and presented using Flow Jo software (Tree Star, San Carlos, CA). 100,000 events are analyzed for each sample.

12.3 Data Collection for Cell Surface T-cell antigen Expression (Dr. Montaner’s Laboratory)

Based on preliminary data suggesting naïve T-cell subsets may decrease in some patients following treatment interruption, these populations will be monitored by whole blood flow cytometry using directly conjugated antibodies. In addition, based on our ability to correlate changes in viral load with T-cell antigen expression, in agreement with published reports 94, we will continue to monitor CD95, HLA-DR and CD28 antigens in the CD4 and CD8 T-cell subsets as markers of activation.

- - 1. Time-points

Refer to Tables 1 through 5 for sample identification in relation to chronology of samples:

- Controls: studied at weeks 0, 20, 40, STI42, STI44, STI46, STI48, STI50 (8 total)
- Experimentals: studied at weeks -2, 0, 2, 4, 8, 12, 17, 19, 23, 27, 36, 38, 42, 46, STI48, STI50, STI32, STI54 (18 total).

12.3.2 Procedure

Whole blood will be stained with five staining combinations using directly conjugated antibodies. Combinations of anti-cell surface antigen antibodies to be used are: CD4/CD28/HLA-DR, CD8/CD28/HLA-DR, CD4/CD45RA/CD45RO, CD4/CD45RA/CD62L, CD8/CD45RA/CD62L and CD3/CD95. The fluorochrome conjugated monoclonal antibodies CD3-PE, CD4-PE, CD8-PE, CD28-FITC, CD45RO-FITC, CD95-FITC are purchased from Pharmingen (San Diego, CA) while, CD4-FITC, CD8-TC, CD45RA-TC and HLA-DR-TC purchased from CalTag (Burlingame, CA). CD62L-PE is obtained from R&D (Minneapolis, MN). The isotype-matched controls IgG1-FITC, IgG1-PE, IgG2a-FITC, IgG2a-PE, IgG2a-TC, and IgG2b-TC, are purchased from Pharmingen. Briefly, 75μl of whole blood is incubated with 7.5μl of FACS blocking buffer (1XPBS, 0.2% BSA, 10% mouse serum, 0.1% sodium azide) for 15 min. at room temperature and stained with the appropriate monoclonal antibody for 30 min. at room temperature. The cells are then lysed with lysis buffer (Becton Dickinson FACS Lyse, Becton Dickinson Immunocytometry Systems, San Jose, CA) for 10 min at room temperature. After being washed twice with FACS washing buffer (1xPBS, 2.5% heat inactivated FBS, 0.1% BSA, 0.02% NaN3) the cells pellets are re-suspended in 500μl of FACS washing buffer and analyzed on a Becton Dickinson FACScalibur flow cytometer using the CellQuest software package for acquisition and analysis. Live-gating on lymphocytes was performed during acquisition of 10,000 events for each condition. Analysis of positively events is done on cells within a manual setting of a lymphogate by forward and side-scatter properties, and thresholds of positive responses are set according to isotype-matched negative controls. Results are expressed as percent positive.

12.4 Data Collection for Thymic Function (Dr. McCune’s Laboratory)

Based on the expected recovery of thymic function in chronically suppressed patients 7, the expected kinetics of circulating T lymphocytes in HIV-1 infection 95., the role of thymic function in generating new immune responses in adults 96,97, and our preliminary observations that treatment interruption can be associated with a decline of the CD4^+^/CD45RA^+^/CD45RO^-^ population in one of five patients followed after therapy interruption, we will monitor thymic output by detection of T-cell receptor excision circles (TREC) following each STI. The presence and number of TRECs in blood cells will be used to infer the activity of thymic production. At a given time point, the presence of TRECs in circulating PBMCs would be consistent with the presence of cells that have recently emigrated from the thymus. If discontinuation of treatment is associated with increased or decreased thymic output, this might be reflected by an increase or decrease, respectively, in the TREC level during the interval of interruption.

12.4.1 Detection of T-cell receptor excision circles

When the V, D, and J gene segments of the T cell receptor (TCR) recombine during the course of thymocyte differentiation, two coding segments are joined in the same transcriptional orientation and the intervening DNA is deleted ^98-100^Precise end-to-end ligation of the recombination signal sequences leads to formation of a circular episome. The presence of these TCR excision circles (TRECs) can be considered diagnostic of recent TCR gene rearrangement and was first used by Cooper and colleagues as a marker of recent thymic emigrants in the chicken 101. In like fashion, assays for the detection of TRECs from the alpha 7 and beta 97 loci of the human TCR gene complex have been devised for the evaluation of thymic function in HIV-1-seropositive and -seronegative adults. Alpha and beta TRECs have been found to be most abundant within circulating naive (CD45RA+CD62L+) CD4+ T cells and the frequency of both alpha TRECs and beta TRECs in circulating PBMCs has been found to be directly related to the abundance of thymic tissue as measured by computed tomography (97and Harris, Jenkins, McCune, unpublished observations)

12.4.2 Time-points

Refer to Tables 1 through 5 for sample identification in relation to chronology of samples:

- Controls: 20 patients studied at weeks 0, 20, 40, STI42, STI44, STI46, STI48, STI50 (8 total)
- Experimentals: 20 patients studied at weeks -2, 0, 2, 4, 8, 12, 17, 19, 23, 27,

36, 38, 42, 46, STI48, STI50, STI32, STI54 (18 total).

12.4.3 Procedure.

The abundance of TRECs in peripheral blood mononuclear cell (PBMC) DNA will be determined by two real-time PCR-based assays which yield complementary information. First, TRECs formed by the rearrangement of the alpha chain of the TCR are determined with specific primers, using Taqman chemistry in a modification of a previously published method 7. These are designed to amplify a unique DNA sequence formed by the joining of delta Rec and psi J alpha regions in the TCR delta locus which are ligated together during the rearrangement of germline DNA early in the process of alpha rearrangement 102. The frequency of alpha TRECS in PBMC samples is calculated with reference to total genomic DNA, as determined in parallel with PCR amplification of a the 5' untranslated region of the human globin gene. Because alpha TRECS are preserved in only one daughter cell during mitosis, the abundance of alpha TRECs per unit PBMC DNA thus reflects indirectly the average number of cell divisions which the lymphocyte population has undergone since formation.

Two potential limitations of the alpha TREC assay induced us to develop an alternative assay for T lymphopoiesis: first, the frequency of alpha TRECs in PBMC is influenced by the presence of non-T-lymphoid elements (monocytes, B cells, natural killer cells), making desirable an assay for TRECs which is normalized to the T cell lineage 96,103. Secondly, alpha TRECs reflect primitive rearrangements which are not related to the post-selection repertoire of true clonotypic rearrangements. For these reasons, we developed a PCR assay for Vbeta2-specific TRECs which is normalized to the abundance of Vbeta2 coding joints. In this assay, we measure concurrently the frequency of Vbeta2 DNA TRECs and the corresponding Vbeta2 coding joints, yielding a direct readout of the average number of mitoses which have occurred since the clonotype specific TCR rearrangement. The real-time PCR assays employ Taqman chemistry, with pre-quenched fluorescent probes which increase in fluorescence intensity as PCR product is generated. The quantity of specific product is determined by two alternative methods: (1) direct quantitation by comparison of PCR signals with copy standards; and (2) calculation of the relative abundance of TREC DNA, by analysing the difference in the number of cycles required for a positive signal for each of the two amplicons (delta Ct analysis). In each method, the abundance of alpha TREC DNA is normalized to that of globin (germline) DNA, and beta TREC DNA is normalized with reference to beta coding joint DNA. In addition, the accuracy of measurement of TREC abundance will be confirmed by the use of replicate determinations in real time PCR and the use of both relative and absolute quantitation methods to measure TREC abundance in blood cells.

Alpha and beta TRECs in peripheral blood cells will be quantitated relative to different signal denominators. We will measure the number of alpha TRECs per 100,000 cells DNA (measured using the single copy β-globin signal) or the number of beta TRECs per Vbeta2 coding joint DNA. Analysis of the relative frequency of each TREC will be accomplished by two methods. First, relative quantitation will be performed by measuring the difference in PCR cycle numbers required to generate an amplification signal from each of the two different amplicons. This analysis of relative abundance, termed ∆Ct analysis, compares the relative abundance of two different templates in a sample. Secondly, to confirm that that ∆Ct analysis is free of confounding factors, we will also perform absolute quantitation of each amplicon using cloned DNA copy number standards. This copy standard approach will be used to validate, on a subset of samples, the routine use of ∆Ct analysis for routine quantitation.

12.5 Data Collection for Viral Genotypes (Dr. Grant’s Laboratory)

Although recent data suggests that a single STI is not associated with genotype resistance, no data is available for the emergence of viral resistance after a sequential series of STIs. As the emergence of drug resistance is a safety concern for participants and for the prevalence of drug resistance in the community 104,105, viral genotypes will be analyzed at baseline and during each STI period in each of the experimental subjects.

12.5.1 Rationale for Genotyping

Viral genetic evidence of resistance selection is one of a secondary endpoints of this study. Genotyping is chosen largely because it is less costly than phenotypic studies. In addition, genotyping generates sequence information that can be used to detect laboratory contamination that is an inherent risk in PCR based assays. Further, genotyping assays have the capacity to distinguish mutational patterns that are difficult to discern from phenotypic assays 106.

12.5.2 Rationale for Genotyping Assay Selection

The TruGene™ Assay (Visible Genetics, Inc) is chosen because in has several advantages over other methods available in Dr. Grant’s laboratory and elsewhere. The advantages of the TruGene assay include (1) Innovations in chemistry and software increase throughput allowing the proposed genotypic analysis to be performed in a timely manner; (2) large portions of the PR and RT reading frames are sequenced, thereby allowing sequence analysis to detect contamination and assess mutation fixation rates; (3) the assay has undergone extensive performance analysis in Dr. Grant’s laboratory as part of preparation for FDA approval; (4) use of dye-primers rather than dye-terminators allows more reliable assessment of the relative proportions of sequence variants in the quasispecies; and (5) Dr. Grant’s laboratory has three research associates who have been thoroughly trained and certified in the performance of this assay. Dye-terminator cycle sequencing (ABI) 107 and sequencing using hybridization to high-density probe arrays (Affymetrix) 108,109 have been used in Dr. Grant’s laboratory, but these assays lack all of the advantages of the TruGene™ assay.

12.5.3 Timepoints

Genotypic assessment will be performed on specimens taken at the time that HAART therapy is maintained before the first STI by culturing latent virus and on plasma samples at the first timepoint when viral load >= 1000 copies/ml is detected during each therapy interruption. In subjects that are found to have wild-type virologic failure, subsequent on-therapy specimens will be analyzed to determine whether drug selection subsequently becomes evident. In subjects who virologically fail with novel or unexpected mutational patterns, sequencing of additional timepoints and phenotypic drug testing will be performed.

12.5.4 Genotyping assay procedures

HIV-1 drug resistance genotyping will be performed using the TruGene™ HIV-1 genotyping assay (Visible Genetics, Toronto, Canada). Briefly, the assay involves reverse transcription, amplification and sequencing of viral RNA extracted from 150 µl of blood plasma (either ACD or EDTA anticoagulated) using columns that reversibly bind RNA to activated silica (Qiagen) The assay can be made still more sensitive through initial centrifugation of plasma to concentration virions 110, and this will be performed if analysis of specimens with viral load less than 1000 RNA copies/ml proves to be warranted in this study.

Dr. Grant’s laboratory performs drug resistance genotyping assays in support of several studies of subjects who have failed protease inhibitor containing regimens 111,112. In the studies that have been completed in Dr. Grant’s laboratory to date have included 30 subjects in whom post-failure sequences clustered with prior sequences in every case. Hence, preliminary data suggests no evidence for contamination as a potential complication at analysis.

# 13.0 STATISTICAL CONSIDERATIONS

- 1. Efficacy Plan and Data Format

The baseline characteristics of the experimental and the control groups will be compared. Continuous variables such as age, peak viral load, and nadir and entry CD4 counts will be summarized by mean, standard deviation, 95% confidence intervals, median, and range. Where continuous variables cannot reasonably be considered to be normally distributed, transformations (e.g., log or square root for skewed data) to achieve a normal distribution will be explored. Categorical variables will be summarized by frequencies. An intent-to-treat analysis will be primary.

13.2 Primary Analysis: Delay of Viral Rebound.

The primary analysis will compare time to viral load rebound between the groups using standard survival time analysis techniques 117. Kaplan-Meier plots will be generated to compare the difference in time to rebound between the groups 120. These plots will also allow us to determine if the difference in time to rebound is statistically significant. However, this technique is limited in that is does not provide a summary statistic describing the magnitude of the difference between the groups. In addition, Kaplan-Meier plots do not permit adjustment for potential confounding variables 120. However, the latter is addressed by our randomization approach. For primary analysis, drop-outs or therapy failures will be considered as rebounded at day 0 of the CTI. Therapy failures in the STI arm are defined as: (1) <50 copies/ml was not achieved within 20 weeks of re-initiated therapy (2) the CD4 cell number declined more than 45% of the baseline CD4 count, (3) participants developed an opportunistic infection even if retaining required CD4 count levels, or (4) the viral load >500,000 copies/ml once with or without development of acute retroviral syndrome as defined by fever, skin lesions, pharyngitis. Therapy failures in the control arm are defined as: (1) viremia between 50 and 999 copies/ml not returning to <50 copies/ml prior to Phase II, and (2) if during the 40 week ART period viral rebound >1000 copies/ml occurs at two consecutive time points.

If we observe difference between groups, Cox proportional hazard modeling will be explored as a technique that is analogous to linear regression with the outcome being time to event and the exposure being whether or not the subject was in the experimental group or the control group 120. A potential limitation to Cox modeling is that it is predicated on the assumption that the hazard of having the event of interest (e.g., viral load rebound) is proportional between the groups over the entire time period. Therefore, before embarking on this analysis, we will test the assumption of proportionality and only proceed given that it is not violated 120. In addition to the simple Cox model including study group as the lone exposure variable, we will explore models that include potential confounding variables. Since the sample size is small, we will explore models that include only one confounding variable in addition to the primary exposure variable per model. The potential confounding variables will be peak viral load prior to therapy, nadir CD4 count and CD4 count at entry. Each of these variables is plausibly related to time to viral load rebound. However, caution will be exercised to not over-fit the models.

- 1. Secondary Analysis

Secondary analyses to be performed in this protocol are centered on enhancing our ability to interpret the effects of STI in regards to specific immune and viral outcomes. These analyses to be performed on selected time-points throughout the study are not intended as the main outcome of this study but as a targeted data collection addressing important secondary questions in regards to STIs.

- - 1. Amplitude of Viral Rebound.

Viral replication magnitude as defined by mean RNA area under the curve will be measured as a secondary outcome at weeks 12 and 20 of the oipen-ended interruption between arms. Another important endpoint in these studies regards the amplitude of viral rebound after successive interruptions. The peak viral rebound during each of the successive STIs will serve this function. These analyses will perform both cross-sectional and longitudinal analyses. The cross-sectional analyses will compare the change between time points for each individual and to determine if the experimental group has had a decrease in their viral loads from prior interruptions. That is, for each individual, the viral load at 4 weeks into the comparison treatment interruption period (CTI) will be compared with the viral load at 4 weeks of the 2^nd^ STI and the 3^rd^ STI respectively. Since these are paired data, we will perform a paired T-test or sign rank test, as appropriate for the underlying distribution of the data 121. In addition, we will perform longitudinal analyses of peak viral rebound during each STI. In order to determine if the subjects in the experimental group exhibit a trend toward lower rebounds with successive interruptions, the viral load after 4 weeks of interruption will be compared within individuals in the experimental group over time. Since only the 2^nd^ and 3^rd^ STIs and the CTI will last long enough (i.e., 4 weeks), they will be the only time points included in this analysis. We will employ GEE in a manner similar to that described above for the longitudinal analysis of the antiviral LPA and CD8 T-cell response data. All analyses will be performed using Stata 5.0 (Stata Corp., College Station, TX) on a Pentium-based PC. All p-values will be two-sided.

- - 1. Analysis of Viral Genotypic Data

The exported consensus sequence at each time-point will be aligned with other sequences from the same study using Clustal W version 1.7 and a multiple sequence alignment file in NBRF format will be exported. A SAS program that reads and translates NBRF files has been developed in Dr. Grant’s laboratory and will be used to generate a SAS databases that will be provided to the study data managers. These databases will be used to identify new mutations that occur relative to baseline, and to correlate new mutations with drug exposure and virological outcomes. Mutations will be classified according to their association with decreased drug susceptibility as defined in the interpretation algorithm established by the Visible Genetics consensus panel. The panel established algorithms based on published data describing correlations between drug susceptibility and viruses derived by point-directed mutagenesis and by amplification from subjects virologically failing antiretroviral therapy. The consensus panel, which includes Dr. Grant, meets at least every 6 months to revise the interpretative rules. In addition, the sequences will be analyzed to determine rate of new mutation fixation at silent, non-silent, and non-silent drug resistance sites. Fixation of mutations at sites known to be selected by drugs will be used as the primary measure of drug-selection. This will be standardized to silent mutation rate if this is found to vary significantly between patients.

The analysis of the emergence of genotypic resistance will help address the safety of this strategy. Genotypic changes for each individual will be scored as present or absent during each STI in the experimentals and controls. For example, if an individual either has wild-type virus at baseline and is found to have resistance mutations during an STI or if that individual starts out with a resistance mutation at baseline and accumulates one or more mutations during an STI, this individual would be counted as having had a genotypic change. Since this is binary data (present or absent), we will compare the proportion of subjects having genotypic resistance changes using the Χ^2^ test 121. Since the experimental group will have many more opportunities to have mutations detected given the more frequent viral rebounds they experience as compared with the controls, these results will bias the study toward finding the emergence of resistance mutations in the experimentals. However, since safety is one of the important end-points of this study and given the limited sample size for detecting safety differences between the groups, oversampling of the experimental group is justified. Furthermore, if no difference between the groups is found regarding the emergence of resistance despite this potential bias, the safety of this strategy would be further strengthened. Of course, interpretation of the safety concerns of finding resistant genotypes as an outcome of STIs will include response to reinitiated therapy. All analyses will be performed using Stata 5.0 (Stata Corp., College Station, TX) on a Pentium-based PC. All p-values will be two-sided.

13.3.3 Lymphoproliferative T-cell Responses.

Recall responses to Candida and HIV-1 will be compared. First, the analysis will describe the data with means, standard deviation, 95% confidence intervals, median, and range. Again, the normality of the data will be explored and transformed where possible. Two groups of analyses will be performed on these data, one cross-sectional and the other longitudinal. The Candida LPA, antiviral LPA and CD8 T-cell response between the groups will be compared in a cross-sectional fashion using Student’s T-test or the Wilcoxon Rank Sum test 121, as applicable, to determine if the strategy increases antiviral LPA and/or CD8 T-cell response. Each of these analyses will be performed for each of the stimuli tested. In the first set of cross-sectional analyses, the LPA data and CD8 T-cell ELISPOT data at the final time point will be compared between the groups. In the second set of cross-sectional analyses, we will compare the change in LPA and ELISPOT data for each individual between their initial measurement and final measurement, using paired T-tests. Of course, these cross-sectional analyses are limited by the fact that they ignore the time points in between the initial and final time points. Biologically, we expect that a trend over time will be occurring in which the experimentals would be experiencing an increase in both antiviral LPA and CD8 T-cell activity over time, not just between initial and final time points. Candida responses are expected to potentially decrease if depleted by HIV-1 replication or sustained if unaffected. We will address this limitation by doing longitudinal analyses.

The longitudinal analyses will also compare the LPA and CD8 T-cell ELISPOT activity between the groups, but will allow for testing for trends in the changes over time. These tests have more statistical power to detect a difference in response than the cross-sectional analyses 121 and would lend further biological credibility of a dose response curve to the sequential STI proposed as the therapeutic intervention in this project. We will use regression analysis to estimate the slope of change of each individual’s antiviral/Candida LPA and CD8 T-cell activity for each stimulus over time. We will then use generalized estimating equations 122-124 to accommodate the correlated nature of the data (i.e., multiple observations over time per individual). The results of these analyses will be a comparison of the trend in immune response over time between the groups. We expect that the activity of the experimental group will be increasing over time, and if the activity of both groups is increasing, that the experimentals will be increasing at a rate greater than that of the controls. An interaction term consisting of the time variable multiplied by the group variable will be the focus of these analyses, since this interaction term will define whether the experimental group has a greater slope over time than the control group.

Since this study is predicated on the fact that we believe that improved immune response will result in improved virological control, another set of analyses will assess the relation between the antiviral/Candida LPA and CD8 T-cell responses in each individual and their time to viral rebound/magnitude. Since neither a clear cutoff for an adequate immune response nor a clinically relevant delay to viral rebound has been established, we will simply assess the correlation between the immune responses and time to viral rebound. We will use either the Pearson correlation coefficient or the Spearman rank-correlation coefficient, depending on the distribution of the immune parameters 121.

13.3.4 Analysis of Cell Surface T-cell antigen Expression

Analysis of T-cell antigen expression will act as an indirect indication of immune activity *in vivo*. Percent population data will be described with means, medians, standard deviations and ranges for both groups. We will graph changes percent positive populations over time for each individual for visual inspection. As we have described, data obtained from both group of patients will be amenable to both cross-sectional and longitudinal analyses. The cross-sectional analysis will compare the percent positive populations between the groups at the final time point using Student’s T-tests or the Wilcoxon rank sum test, depending on the distribution of the data 121. The longitudinal analysis will address the potential dose-response nature of the data. For this analysis, since the amount of percent positive cells might be different while on HAART versus off HAART, we will perform three sets of analyses of the experimental group. The first analysis of the experimentals will include only those time points on HAART (i.e., between STIs) to determine if, over time, the amount of cells in each subset measured is increasing. The second analysis will include only those time points off HAART (i.e., during STIs). The third analysis will include all time points and will determine if the relative percent changes throughout the study period, irrespective of being on or off HAART. Since our observations already indicate a potential positive (i.e., HLA-DR), negative (i.e., CD45RA), or no effect (i.e., CD4) relationship between expression of specific lymphocyte antigens and viral load, we will perform another set of analyses to assess the relation between total percent populations in each individual and viral load. Since a clinically relevant change in markers other than CD4 has not been established, we will simply assess the correlation between levels of percent positive T-cell subsets and viral rebound. We will use either the Pearson correlation coefficient or the Spearman rank-correlation coefficient, depending on the distribution of the immune parameters 121.All analyses will be performed using Stata 5.0 (Stata Corp., College Station, TX) on a Pentium-based PC. All p-values will be two-sided. We expect to characterize changes in naïve T-cell subsets in relation to treatment interruption. We also expect to show a correlation between HLA-DR expression on T-cell subsets to be associated with viral load.

13.3.5 Analysis of Thymic Function

The impact of the STIs on the relative abundance of recent thymic emigrants is currently unknown and this study will allow us to determine this effect. For purposes of analysis, TREC signal will be normalized with the data acquired on the same day for lymphocyte numbers per microliter (CBC Differential) and the percent CD45RA+/CD62L+ obtained on the lymphocyte populations (See Section C.3.1). As we have described, data obtained from both groups of patients will be amenable to both cross-sectional and longitudinal analyses. The cross-sectional analysis will compare the relative abundance of TREC signal between the groups at the final time point using Student’s T-tests or the Wilcoxon rank sum test, depending on the distribution of the data 121. The longitudinal analysis will address the potential dose-response nature of the data. For this analysis, since the effect on relative abundance of TREC signal might be different while on HAART versus off HAART, we will perform three sets of analyses of the experimental group. The first analysis of the experimentals will include only those time points on HAART (i.e., between STIs) to determine if, over time, the relative abundance of TRECs is increasing. The second analysis will include only those time points off HAART (i.e., during STIs). The third analysis will include all time points and will determine if the relative abundance of TRECs increases throughout the study period, irrespective of being on or off HAART. All analyses will be performed using Stata 5.0 (Stata Corp., College Station, TX) on a Pentium-based PC. All p-values will be two-sided.

We expect to show that STI will have an impact on thymic function, observed as either (a) an increase (relative to baseline) in the level of circulating TRECs over the total interval of the on/off cycles (reflecting a potentially beneficial effect of treatment interruption); or (b) an increase in the level of circulating TRECs during periods of treatment, relative to those levels found during periods of treatment interruption (reflecting a potentially adverse effect of treatment interruption). Concerning the latter outcome, a potential limitation of this assay at analysis is the multiple reasons that could result in a decrease of TREC levels. TREC levels could appear to decrease if recent thymic emigrants (RTEs) proliferated, died, or left the circulation more rapidly; alternatively, selective movement of non-RTEs (i.e., more mature T cells) into the peripheral circulation could result in an apparent decrease in TREC frequency. Conceivably, each of these events could occur upon cessation and/or resumption of therapy. On the other hand, the interpretation of an increase in circulating TREC levels is less problematic: absent increased thymic output of RTEs, such an increase would require that RTEs accumulate in the peripheral circulation relative to other (non-RTE) cell subpopulations. Within the time frame of the experiments in this proposal, such differential accumulation is possible but biologically implausible.

14.0 DATA SAFETY AND MONITORING BOARD GUIDELINES

Oversight of safety as a primary aim of this study will be formally monitored once a year by a Safety Monitoring Board whose membership will include: Dr. Harvey Friedman (chair), Dr. Rosalie Pepe, Ms. Julie Davis (community representative), and Ms. Ronda Goldfein (Lawyer). Dr. Jay Kostman will be a non-voting member of the DSMB as the principal clinical investigator of the protocol.

Safety during the three time-specified STIs (2, 4, 6 weeks) and final comparison treatment interruption (CTI) (open-ended) periods will be monitored by CD4 counts, clinical symptoms, and VL. Treatment will be re-initiated before indicated by study design if: (a) CD4 count declines greater than 45% CD4 count from baseline (43.2% represents a 20% higher degree of CD4 decline than what was observed in the observational cohort following treatment withdrawal), (b) clinical progression of HIV disease, (c) onset of acute retroviral syndrome, or (d) viral load rises above 500,000 copies/ml. Subjects experiencing adverse effects a-c during an STI will be discontinued from additional STI’s at the discretion of the DSMB. For the latter patients, their time to rebound while off therapy during that STI will be used as the CTI data for analysis with the remainder subjects in the STI group. The latter will avoid bias for positive responders at analysis. Patients during the CTI period that experience a-c will be exempt from the three-week period of viral load measurements before reinitiating therapy. Any patient refusing to reinitiate therapy at indicated times will not be used in evaluating safety outcomes. Subjects who do not show greater than 0.5 log reduction in viral load by 6 weeks of re-initiated therapy or who do not achieve <50 copies/ml by 20 weeks under therapy will be offered GART testing.

The risks identified with this intervention are: (a) decline greater than 45% in CD4 cell percent from that present at the baseline for the study, (b) emergence of viral resistance to treatment, (c) clinical progression of disease in association with decrease in CD4 percent. Therefore, the DSMB will formally monitor changes in CD4 cell count/percent and HIV RNA for the first year of the study. Attention will be particularly be paid to CD4 cell decline during the STI, and to recovery of CD4 cells and viral load response following reinitiation of therapy. If greater than 50% of individuals randomized to the STI group have less than a 25% recovery of CD4 cells following the reinitiation of treatment then accrual may be temporarily stopped. Similarly, if greater than 50% of individuals randomized to the STI group do not achieve viral suppression after re-initiation of therapy, prior to the next interruption, then accrual may be temporarily stopped. The protocol will be considered for termination if 7 subjects (33%) in the STI arm of 26 experience a stable decline of CD4 count to lower than 200 cells/μl under reinitiated therapy or are unable to achieve <50 copies/ml by 20 weeks of re-initiated therapy in spite of adherence.

If the DSMB decides at any time to stop accrual, CD4 cell count and HIV RNA responses will continue to be monitored on a monthly basis to determine whether accrual can be safely resumed based on trends in these variables.

14.1 Main Safety Outcome End-points

- Change in baseline CD4 percent. Endpoint is a greater than 45% decline from baseline.
- Lack of viral control at 20 weeks of reinitiated treatment. Endpoint is greater than 50 copies/ml with over 90% adherence over 20 weeks.

15.0 COMPENSATION, INSURANCE AND INDEMNITY

The Sponsor is liable, in accordance with the local law, for any damage directly related to the investigator’s and subject’s participation in the study.

Compensation will be given to each subject at $25.00 per visit to cover parking and transportation costs.

# 16.0 RETENTION OF RECORDS

Source documents and related study records, including CRFs, will be maintained at the study site in accordance with all international and federal guidelines. In general, records will be maintained for a minimum of 15 years after study completion. The Principal Investigator may withdraw from the responsibility to maintain records and transfer custody of the records to another person who will accept responsibility for them. Notice of this transfer must be submitted and approved by the IRB prior to such action.

17.0 CONFIDENTIALITY OF INFORMATION

It is understood that information derived from the study will be used for publication. No individual investigator may independently publish until the group has published the study results in aggregate. The Scientific Committee will designate a Writing Committee to prepare the publication of the main study results. All publications resulting from the clinical trial must be in a form that protects patient confidentiality.

# 18.0 REFERENCES

1 Finzi, D., Hermankova, M., Pierson, T. *et al*. (1997)Identification of a reservoir for HIV-1 in patients on highly active antiretroviral therapy [see comments] *Science* 278(5341), 1295-1300

2 Finzi, D., Blankson, J., Siliciano, J.D. *et al*. (1999)Latent infection of CD4+ T cells provides a mechanism for lifelong persistence of HIV-1, even in patients on effective combination therapy *Nat Med* 5(5), 512-517

3 Furtado, M.R., Callaway, D.S., Phair, J.P. *et al*. (1999)Persistence of HIV-1 transcription in peripheral-blood mononuclear cells in patients receiving potent antiretroviral therapy [see comments] *N Engl J Med* 340(21), 1614-1622

4 Jubault, V., Burgard, M., Le Corfec, E. *et al*. (1998)High rebound of plasma and cellular HIV load after discontinuation of triple combination therapy [letter] *Aids* 12(17), 2358-2359

5 Zhang, L., Ramratnam, B., Tenner-Racz, K. *et al*. (1999)Quantifying residual HIV-1 replication in patients receiving combination antiretroviral therapy [see comments] *N Engl J Med* 340(21), 1605-1613

6 Greenberg, P.D. and Riddell, S.R. (1999)Deficient cellular immunity--finding and fixing the defects *Science* 285(5427), 546-551

7 Douek, D.C., McFarland, R.D., Keiser, P.H. *et al*. (1998)Changes in thymic function with age and during the treatment of HIV infection [see comments] *Nature* 396(6712), 690-695

8 Pantaleo, G. (1997)How immune-based interventions can change HIV therapy [see comments] *Nat Med* 3(5), 483-486

9 Wilson, C.C., Olson, W.C., Tuting, T. *et al*. (1999)HIV-1-specific CTL responses primed in vitro by blood-derived dendritic cells and Th1-biasing cytokines *J Immunol* 162(5), 3070-3078

10 Schmitz, J.E., Kuroda, M.J., Santra, S. *et al*. (1999)Control of viremia in simian immunodeficiency virus infection by CD8+ lymphocytes *Science* 283(5403), 857-860

11 Rinaldo, C., Huang, X.L., Fan, Z.F. *et al*. (1995)High levels of anti-human immunodeficiency virus type 1 (HIV-1) memory cytotoxic T-lymphocyte activity and low viral load are associated with lack of disease in HIV-1-infected long-term nonprogressors *J Virol* 69(9), 5838-5842

12 Harrer, T., Harrer, E., Kalams, S.A. *et al*. (1996)Cytotoxic T lymphocytes in asymptomatic long-term nonprogressing HIV-1 infection. Breadth and specificity of the response and relation to in vivo viral quasispecies in a person with prolonged infection and low viral load *J Immunol* 156(7), 2616-2623

13 Cao, Y., Qin, L., Zhang, L., Safrit, J. and Ho, D.D. (1996)Characterization of long-term survivors of human immunodeficiency virus type 1 infection [retracted in Immunol Lett 1998 Nov;64(1):following 55] *Immunol Lett* 51(1-2), 7-13

14 Cao, Y., Qin, L., Zhang, L., Safrit, J. and Ho, D.D. (1995)Virologic and immunologic characterization of long-term survivors of human immunodeficiency virus type 1 infection [see comments] *N Engl J Med* 332(4), 201-208

15 Koup, R.A., Safrit, J.T., Cao, Y. *et al*. (1994)Temporal association of cellular immune responses with the initial control of viremia in primary human immunodeficiency virus type 1 syndrome *J Virol* 68(7), 4650-4655

16 Bollinger, R.C., Egan, M.A., Chun, T.W., Mathieson, B. and Siliciano, R.F. (1996)Cellular immune responses to HIV-1 in progressive and non-progressive infections *Aids* 10(Suppl A), S85-96

17 Pontesilli, O., Carotenuto, P., Kerkhof-Garde, S.R. *et al*. (1999)Lymphoproliferative response to HIV type 1 p24 in long-term survivors of HIV type 1 infection is predictive of persistent AIDS-free infection [In Process Citation] *AIDS Res Hum Retroviruses* 15(11), 973-981

18 Dyer, W.B., Ogg, G.S., Demoitie, M.A. *et al*. (1999)Strong human immunodeficiency virus (HIV)-specific cytotoxic T- lymphocyte activity in Sydney Blood Bank Cohort patients infected with nef-defective HIV type 1 *J Virol* 73(1), 436-443

19 Rowland-Jones, S. (1999)Long-term non-progression in HIV infection: clinico pathological issues *J Infect* 38(2), 67-70

20 Rowland-Jones, S.L., Dong, T., Dorrell, L. *et al*. (1999)Broadly cross-reactive HIV-specific cytotoxic T-lymphocytes in highly- exposed persistently seronegative donors *Immunol Lett* 66(1-3), 9-14

21 Fowke, K.R., Dong, T., Rowland-Jones, S.L. *et al*. (1998)HIV type 1 resistance in Kenyan sex workers is not associated with altered cellular susceptibility to HIV type 1 infection or enhanced beta-chemokine production *AIDS Res Hum Retroviruses* 14(17), 1521-1530

22 Rosenberg, E.S., Billingsley, J.M., Caliendo, A.M. *et al*. (1997)Vigorous HIV-1-specific CD4+ T cell responses associated with control of viremia [see comments] *Science* 278(5342), 1447-1450

23 Walker, B.D., Rosenberg, E.S., Hay, C.M., Basgoz, N. and Yang, O.O. (1998)Immune control of HIV-1 replication *Adv Exp Med Biol* 452, 159-167

24 Merrill, D.P., Martinez-Picado, J., Tremblay, C. *et al*. (1999)Improved CD4 lymphocyte outgrowth in response to effective antiretroviral therapy *J Infect Dis* 179(2), 345-351

25 Gorochov, G., Neumann, A.U., Kereveur, A. *et al*. (1998)Perturbation of CD4+ and CD8+ T-cell repertoires during progression to AIDS and regulation of the CD4+ repertoire during antiviral therapy [see comments] *Nat Med* 4(2), 215-221

26 Markowitz, M., Vesanen, M., Tenner-Racz, K. *et al*. (1999)The effect of commencing combination antiretroviral therapy soon after human immunodeficiency virus type 1 infection on viral replication and antiviral immune responses [published erratum appears in J Infect Dis 1999 May;179(5):1315] *J Infect Dis* 179(3), 527-537

27 Autran, B., Carcelain, G., Li, T.S. *et al*. (1997)Positive effects of combined antiretroviral therapy on CD4+ T cell homeostasis and function in advanced HIV disease [see comments] *Science* 277(5322), 112-116

28 Komanduri, K.V., Viswanathan, M.N., Wieder, E.D. *et al*. (1998)Restoration of cytomegalovirus-specific CD4+ T-lymphocyte responses after ganciclovir and highly active antiretroviral therapy in individuals infected with HIV-1 *Nat Med* 4(8), 953-956

29 Lederman, M.M., Connick, E., Landay, A. *et al*. (1998)Immunologic responses associated with 12 weeks of combination antiretroviral therapy consisting of zidovudine, lamivudine, and ritonavir: results of AIDS Clinical Trials Group Protocol 315 *J Infect Dis* 178(1), 70-79

30 Li, T.S., Tubiana, R., Katlama, C. *et al*. (1998)Long-lasting recovery in CD4 T-cell function and viral-load reduction after highly active antiretroviral therapy in advanced HIV-1 disease [see comments] *Lancet* 351(9117), 1682-1686

31 Mezzaroma, I., Carlesimo, M., Pinter, E. *et al*. (1999)Long-term evaluation of T-cell subsets and T-cell function after HAART in advanced stage HIV-1 disease [In Process Citation] *Aids* 13(10), 1187-1193

32 O'Sullivan, C.E., Drew, W.L., McMullen, D.J. *et al*. (1999)Decrease of Cytomegalovirus Replication in Human Immunodeficiency Virus Infected-Patients after Treatment with Highly Active Antiretroviral Therapy *J Infect Dis* 180(3), 847-849

33 Pontesilli, O., Kerkhof-Garde, S., Notermans, D.W. *et al*. (1999)Functional T cell reconstitution and human immunodeficiency virus-1- specific cell-mediated immunity during highly active antiretroviral therapy *J Infect Dis* 180(1), 76-86

34 Haase, A.T. (1999)Population biology of HIV-1 infection: viral and CD4+ T cell demographics and dynamics in lymphatic tissues *Annu Rev Immunol* 17, 625-656

35 Connors, M., Kovacs, J.A., Krevat, S. *et al*. (1997)HIV infection induces changes in CD4+ T-cell phenotype and depletions within the CD4+ T-cell repertoire that are not immediately restored by antiviral or immune-based therapies [see comments] *Nat Med* 3(5), 533-540

36 Berman, P.W. (1998)Development of bivalent rgp120 vaccines to prevent HIV type 1 infection *AIDS Res Hum Retroviruses* 14 Suppl 3, S277-289

37 Francis, D.P., Gregory, T., McElrath, M.J. *et al*. (1998)Advancing AIDSVAX to phase 3. Safety, immunogenicity, and plans for phase 3 *AIDS Res Hum Retroviruses* 14 Suppl 3, S325-331

38 Ogg, G.S., Jin, X., Bonhoeffer, S. *et al*. (1998)Quantitation of HIV-1-specific cytotoxic T lymphocytes and plasma load of viral RNA *Science* 279(5359), 2103-2106

39 Ogg, G.S., Jin, X., Bonhoeffer, S. *et al*. (1999)Decay kinetics of human immunodeficiency virus-specific effector cytotoxic T lymphocytes after combination antiretroviral therapy *J Virol* 73(1), 797-800

40 Pitcher, C.J., Quittner, C., Peterson, D.M. *et al*. (1999)HIV-1-specific CD4+ T cells are detectable in most individuals with active HIV-1 infection, but decline with prolonged viral suppression [see comments] *Nat Med* 5(5), 518-525

41 Lisziewicz, J., Rosenberg, E., Lieberman, J. *et al*. (1999)Control of HIV despite the discontinuation of antiretroviral therapy [letter] *N Engl J Med* 340(21), 1683-1684

42 Ortiz, G.M., Nixon, D.F., Trkola, A. *et al*. (1999)HIV-1-specific immune responses in subjects who temporarily contain virus replication after discontinuation of highly active antiretroviral therapy [see comments] *J Clin Invest* 104(6), R13-18

43 Hammer, S.M., Squires, K.E., Hughes, M.D. *et al*. (1997)A controlled trial of two nucleoside analogues plus indinavir in persons with human immunodeficiency virus infection and CD4 cell counts of 200 per cubic millimeter or less. AIDS Clinical Trials Group 320 Study Team [see comments] *N Engl J Med* 337(11), 725-733

44 Mellors, J.W., Rinaldo, C.R., Jr., Gupta, P. *et al*. (1996)Prognosis in HIV-1 infection predicted by the quantity of virus in plasma [see comments] [published erratum appears in Science 1997 Jan 3;275(5296):14] *Science* 272(5265), 1167-1170

45 Candotti, D., Costagliola, D., Joberty, C. *et al*. (1999)Status of long-term asymptomatic HIV-1 infection correlates with viral load but not with virus replication properties and cell tropism. French ALT Study Group *J Med Virol* 58(3), 256-263

46 O'Brien, T.R., Blattner, W.A., Waters, D. *et al*. (1996)Serum HIV-1 RNA levels and time to development of AIDS in the Multicenter Hemophilia Cohort Study [see comments] *Jama* 276(2), 105-110

47 (1998)Report of the NIH Panel To Define Principles of Therapy of HIV Infection *Ann Intern Med* 128(12 Pt 2), 1057-1078

48 Gulick, R.M. (1998)HIV treatment strategies: planning for the long term [editorial; comment] [published erratum appears in JAMA 1998 Jun 3;279(21):1702] [see comments] *Jama* 279(12), 957-959

49 Arya, S.C. (1998)Antiretroviral therapy in countries with low health expenditure [letter; comment] *Lancet* 351(9113), 1433-1434

50 Stephenson, J. (1999)AIDS researchers target poor adherence [news] *Jama* 281(12), 1069

51 Saag, M.S. and Kilby, J.M. (1999)HIV-1 and HAART: a time to cure, a time to kill [news; comment] *Nat Med* 5(6), 609-611

52 Lo, J.C., Mulligan, K., Tai, V.W., Algren, H. and Schambelan, M. (1998)"Buffalo hump" in men with HIV-1 infection [see comments] *Lancet* 351(9106), 867-870

53 Miller, K.D., Jones, E., Yanovski, J.A. *et al*. (1998)Visceral abdominal-fat accumulation associated with use of indinavir [see comments] *Lancet* 351(9106), 871-875

54 Carr, A., Samaras, K., Burton, S. *et al*. (1998)A syndrome of peripheral lipodystrophy, hyperlipidaemia and insulin resistance in patients receiving HIV protease inhibitors *Aids* 12(7), F51-58

55 Roth, V.R., Kravcik, S. and Angel, J.B. (1998)Development of cervical fat pads following therapy with human immunodeficiency virus type 1 protease inhibitors [see comments] *Clin Infect Dis* 27(1), 65-67

56 Kotler, D., Rosenbaum, K., Wang, J. and al., e. (June-July 1998) in *12th World AIDS Conference*.

57 Gervasoni, D., Ridolfo, A. and Trifiro, G. (September 1998) in *38th Annual Interscience Conference on Antimicrobial Agents and Chemotherapy*.

58 Dong, K., Flynn, M., Dickinson, B. and al, e. (June-July 1998) in *12th World AIDS Conference*.

59 Rosenberg, H., Mulder, J., Sepkowitz, K. and MF, G. (Febuary, 1998) in *5th Conference on Human Retroviruses and Opportunistic Infections*.

60 Melnikow, J. and Kiefe, C. (1994)Patient compliance and medical research: issues in methodology *J Gen Intern Med* 9(2), 96-105

61 Morisky, D.E., Green, L.W. and Levine, D.M. (1986)Concurrent and predictive validity of a self-reported measure of medication adherence *Med Care* 24(1), 67-74

62 Norell, S.E. (1984)Methods in assessing drug compliance *Acta Med Scand Suppl* 683, 35-40

63 Gordis, L. (1979) in *Compliance in Health Care* (Haynes R, T.D., Sackett D, ed.), pp. 21, Johns Hopkins University Press.

64 Cramer, J. (1991) in *Patient Compliance in Medical Practice and Clinical Trials* (Cramer J, S.B., ed.), pp. 3, Raven Press.

65 Cromer, B.A., Steinberg, K., Gardner, L., Thornton, D. and Shannon, B. (1989)Psychosocial determinants of compliance in adolescents with iron deficiency *Am J Dis Child* 143(1), 55-58

66 Rudd, P., Byyny, R.L., Zachary, V. *et al*. (1989)The natural history of medication compliance in a drug trial: limitations of pill counts [see comments] *Clin Pharmacol Ther* 46(2), 169-176

67 Pullar, T., Kumar, S., Tindall, H. and Feely, M. (1989)Time to stop counting the tablets? [see comments] *Clin Pharmacol Ther* 46(2), 163-168

68 Cheung, R., Dickins, J., Nicholson, P.W. *et al*. (1988)Compliance with anti-tuberculous therapy: a field trial of a pill-box with a concealed electronic recording device *Eur J Clin Pharmacol* 35(4), 401-407

69 Cheung, R., Sullens, C.M., Seal, D. *et al*. (1988)The paradox of using a 7 day antibacterial course to treat urinary tract infections in the community *Br J Clin Pharmacol* 26(4), 391-398

70 Cramer, J., Vachon, L., Desforges, C. and Sussman, N.M. (1995)Dose frequency and dose interval compliance with multiple antiepileptic medications during a controlled clinical trial *Epilepsia* 36(11), 1111-1117

71 Deeks, S.G. and Volberding, P.A. (1997)HIV-1 protease inhibitors *AIDS Clin Rev* , 145-185

72 Molla, A., Korneyeva, M., Gao, Q. *et al*. (1996)Ordered accumulation of mutations in HIV protease confers resistance to ritonavir *Nat Med* 2(7), 760-766

73 Weis, S.E., Slocum, P.C., Blais, F.X. *et al*. (1994)The effect of directly observed therapy on the rates of drug resistance and relapse in tuberculosis [see comments] *N Engl J Med* 330(17), 1179-1184

74 Kent, J.H. (1993)The epidemiology of multidrug-resistant tuberculosis in the United States *Med Clin North Am* 77(6), 1391-1409

75 Cuneo, W.D. and Snider, D.E., Jr. (1989)Enhancing patient compliance with tuberculosis therapy *Clin Chest Med* 10(3), 375-380

76 Cramer, J.A., Mattson, R.H., Prevey, M.L., Scheyer, R.D. and Ouellette, V.L. (1989)How often is medication taken as prescribed? A novel assessment technique [published erratum appears in JAMA 1989 Sep 15;262(11):1472] [see comments] *Jama* 261(22), 3273-3277

77 Kruse, W. and Weber, E. (1990)Dynamics of drug regimen compliance--its assessment by microprocessor- based monitoring *Eur J Clin Pharmacol* 38(6), 561-565

78 Rudd, P., Ahmed, S., Zachary, V. and Barton, C. (1992)Compliance with medication timing: implications from a medication trial for drug development and clinical practice *J Clin Res and Pharmacoepidemiology* 6, 15-27

79 Cramer, J.A. (1995)Microelectronic systems for monitoring and enhancing patient compliance with medication regimens *Drugs* 49(3), 321-327

80 Cramer, J.A., Scheyer, R.D. and Mattson, R.H. (1990)Compliance declines between clinic visits [see comments] *Arch Intern Med* 150(7), 1509-1510

81 Brun, J. (1994)Patient compliance with once-daily and twice-daily oral formulations of 5-isosorbide mononitrate: a comparative study [published erratum appears in J Int Med Res 1994 Nov-Dec;22(6):350] *Journal of International Medical Research* 22(5), 266-272

82 Matsui, D., Hermann, C., Klein, J. *et al*. (1994)Critical comparison of novel and existing methods of compliance assessment during a clinical trial of an oral iron chelator *J Clin Pharmacol* 34(9), 944-949

83 Olivieri, N.F., Matsui, D., Hermann, C. and Koren, G. (1991)Compliance assessed by the Medication Event Monitoring System *Arch Dis Child* 66(12), 1399-1402

84 Neumann, A.U., Tubiana, R., Calvez, V. *et al*. (1999)HIV-1 rebound during interruption of highly active antiretroviral therapy has no deleterious effect on reinitiated treatment. Comet Study Group [In Process Citation] *Aids* 13(6), 677-683

85 Dupont, W.D. and Plummer, W.D., Jr. (1990)Power and sample size calculations. A review and computer program *Control Clin Trials* 11(2), 116-128

86 Bailer, R., Holloway, A., Anthony, R. *et al*. (1998)Deficiency of IL-13 and IFN-γ secretion in HIV infected individuals contributes to overall immune dysfunction *5th Conf. Retro. and Opportun. Infect.* p193 (abstract no. 602), Feb 1-5

87 Weinberg, A., Betensky, R.A., Zhang, L. and Ray, G. (1998)Effect of shipment, storage, anticoagulant, and cell separation on lymphocyte proliferation assays for human immunodeficiency virus- infected patients *Clin Diagn Lab Immunol* 5(6), 804-807

88 Lalvani, A., Brookes, R., Hambleton, S. *et al*. (1997)Rapid effector function in CD8+ memory T cells *J Exp Med* 186(6), 859-865

89 Larsson, M. and al., e. (1999)A recombinant vaccinia virus based ELISPOT assay detects high frequencies of Pol-specific CD8 T cells in HIV-1-positive individuals *AIDS* 13, 767-777

90 Kuroda, M.J., Schmitz, J.E., Barouch, D.H. *et al*. (1998)Analysis of Gag-specific cytotoxic T lymphocytes in simian immunodeficiency virus-infected rhesus monkeys by cell staining with a tetrameric major histocompatibility complex class I-peptide complex *J Exp Med* 187(9), 1373-1381

91 Altman, J.D., Moss, P.A.H., Goulder, P.J.R. *et al*. (1996)Phenotypic analysis of antigen-specific T lymphocytes [published erratum appears in Science 1998 Jun 19;280(5371):1821] *Science* 274(5284), 94-96

92 Kern, F., Surel, I.P., Brock, C. *et al*. (1998)T-cell epitope mapping by flow cytometry *Nat Med* 4(8), 975-978

93 Murali-Krishna, K., Altman, J.D., Suresh, M. *et al*. (1998)Counting antigen-specific CD8 T cells: a reevaluation of bystander activation during viral infection *Immunity* 8(2), 177-187

94 Autran, B., Carcelaint, G., Li, T.S. *et al*. (1999)Restoration of the immune system with anti-retroviral therapy *Immunol Lett* 66(1-3), 207-211

95 Hellerstein, M., Hanley, M.B., Cesar, D. *et al*. (1999)Directly measured kinetics of circulating T lymphocytes in normal and HIV-1-infected humans [see comments] *Nat Med* 5(1), 83-89

96 Jamieson, B.D., Douek, D.C., Killian, S. *et al*. (1999)Generation of functional thymocytes in the human adult *Immunity* 10(5), 569-575

97 Poulin, J., Viswanathan, M., Komanduri, K. *et al*. (1999)Direct evidence for thymic function in adult humans *J. Exp. Med.* 190, 479-486

98 Fujimoto, S. and Yamagishi, H. (1987)Isolation of an excision product of T-cell receptor alpha-chain gene rearrangements [published erratum appears in Nature 1987 Jun 4- 10;327(6121):439] *Nature* 327(6119), 242-243

99 McCormack, W.T., Tjoelker, L.W., Carlson, L.M. *et al*. (1989)Chicken IgL gene rearrangement involves deletion of a circular episome and addition of single nonrandom nucleotides to both coding segments *Cell* 56(5), 785-791

100 Okazaki, K., Davis, D.D. and Sakano, H. (1987)T cell receptor beta gene sequences in the circular DNA of thymocyte nuclei: direct evidence for intramolecular DNA deletion in V-D-J joining *Cell* 49(4), 477-485

101 Kong, F., Chen, C.H. and Cooper, M.D. (1998)Thymic function can be accurately monitored by the level of recent T cell emigrants in the circulation *Immunity* 8(1), 97-104

102 Takeshita, S., Toda, M. and Yamagishi, H. (1989)Excision products of the T cell receptor gene support a progressive rearrangement model of the alpha/delta locus *Embo J* 8(11), 3261-3270

103 Zhang, L., Lewin, S.R., Markowitz, M. *et al*. (1999)Measuring recent thymic emigrants in blood of normal and HIV-1-infected individuals before and after effective therapy *J Exp Med* 190(5), 725-732

104 Grant, R., Hecht,F, Petropoulos, C, Dillon,B, Chesney, M, Tian, H, Hellmann, N & Kahn, J. (1998) in *2nd International Workshop on HIV Drug Resistance and Treatment Strategies*, International Medical Press.

105 Hecht, F.M., Grant, R.M., Petropoulos, C.J. *et al*. (1998)Sexual transmission of an HIV-1 variant resistant to multiple reverse- transcriptase and protease inhibitors [see comments] *N Engl J Med* 339(5), 307-311

106 Yerly, S., Rakik, A., De Loes, S.K. *et al*. (1998)Switch to unusual amino acids at codon 215 of the human immunodeficiency virus type 1 reverse transcriptase gene in seroconvertors infected with zidovudine-resistant variants *J Virol* 72(5), 3520-3523

107 Winters, M.A., Shafer, R.W., Jellinger, R.A. *et al*. (1997)Human immunodeficiency virus type 1 reverse transcriptase genotype and drug susceptibility changes in infected individuals receiving dideoxyinosine monotherapy for 1 to 2 years *Antimicrob Agents Chemother* 41(4), 757-762

108 Hecht, F., Kahn, JO, Dillon, B, Chesney, M & Grant, RM (1998) in *Int Conf AIDS*.

109 Gunthard, H.F., Wong, J.K., Ignacio, C.C., Havlir, D.V. and Richman, D.D. (1998)Comparative performance of high-density oligonucleotide sequencing and dideoxynucleotide sequencing of HIV type 1 pol from clinical samples *AIDS Res Hum Retroviruses* 14(10), 869-876

110 Lloyd, R., Schuuman, R., Stang, H. *et al*. (1999)Accuracy and reproducibility of ultra-low genotyping *Antiviral Therapy* 4, 35

111 Deeks, S.G., Grant, R.M., Beatty, G.W. *et al*. (1998)Activity of a ritonavir plus saquinavir-containing regimen in patients with virologic evidence of indinavir or ritonavir failure *Aids* 12(10), F97-102

112 Deeks, S.G., Hellmann, N.S., Grant, R.M. *et al*. (1999)Novel four-drug salvage treatment regimens after failure of a human immunodeficiency virus type 1 protease inhibitor-containing regimen: antiviral activity and correlation of baseline phenotypic drug susceptibility with virologic outcome *J Infect Dis* 179(6), 1375-1381

113 Chun, T.W., Stuyver, L., Mizell, S.B. *et al*. (1997)Presence of an inducible HIV-1 latent reservoir during highly active antiretroviral therapy *Proc Natl Acad Sci U S A* 94(24), 13193-13197

114 Wong, J.K., Hezareh, M., Gunthard, H.F. *et al*. (1997)Recovery of replication-competent HIV despite prolonged suppression of plasma viremia [see comments] *Science* 278(5341), 1291-1295

115 Staprans, S., Marlowe, N., Glidden, D. *et al*. (1999)Time course of cerebrospinal fluid responses to antiretroviral therapy: evidence for variable compartmentalization of infection *Aids* 13(9), 1051-1061

116 Staprans, S.I., Hamilton, B.L., Follansbee, S.E. *et al*. (1995)Activation of virus replication after vaccination of HIV-1-infected individuals *J Exp Med* 182(6), 1727-1737

117 Piantadosi, S. (1997) *Clinical Trials: A Methodologic Perspective*, John Wiley & Sons

118 Gross, R., Friedman, H., Bilker, W. and Strom, B. (1999)Impact of Nelvinavir pill-taking behavior on suppression of HIV in protease inhibitor naive subjects *National Meeting of General Clinical Research Centers* Washington, D.C.

119 Hanley, J.A. and McNeil, B.J. (1982)The meaning and use of the area under a receiver operating characteristic (ROC) curve *Radiology* 143(1), 29-36

120 Collett, D. (1994) *Modelling Survival Data in Medical Research*, Chapman & Hall

121 Armitage, P. and Berry, G. (1987) *Statistical Methods in Medical Research*, Blackwell Scientific Publications

122 McCullagh, P. and Nelder, J. (1989) *Generalized Linear Models*, Chapman Hall

123 Zeger, S.L. and Liang, K.Y. (1986)Longitudinal data analysis for discrete and continuous outcomes *Biometrics* 42(1), 121-130

124 Zeger, S.L. and Liang, K.Y. (1992)An overview of methods for the analysis of longitudinal data *Stat Med* 11(14-15), 1825-1839.

## 19.0 APPENDICES

Appendix 1. Declaration of Helsinki

Appendix 2. Informed Consent

Appendix 3. Performance Index

Appendix 4. Case Report Forms

Appendix 5 Study Flowchart

**Appendix 1.**

**Declaration of Helsinki**

Recommendations guiding medical doctors in biomedical research involving human subjects adopted by the 18^th^ World Medical Assembly, Helsinki, Finland, June 1964, amended by the 29^th^ World Medical Assembly, Tokyo, Japan, October 1975, the 35^th^ World Medical Assembly, Venice, Italy, October 1983, the 41^st^ World Medical Assembly, Hong Kong, September 1989, and the Somerset West revision of 1996.

Introduction

It is the mission of the physician to safeguard the health of the people. His or her knowledge and conscience are dedicated to the fulfillment of this mission.

The Declaration of Geneva of the World Medical Association binds the physician with the words, "The Health of my patient will be my first consideration," and the International Code of Medical Ethics declares that, "A physician shall act only in the patient's interest when providing medical care which might have the effect of weakening the physical and mental condition of the patient. "

The purpose of biomedical research involving human subjects must be to improve diagnostic, therapeutic and prophylactic procedures and the understanding of the aetiology and pathogenesis of disease.

In current medical practice most diagnostic, therapeutic or prophylactic procedures involve hazards. This applies especially to biomedical research.

Medical progress is based on research which ultimately must rest in part on experimentation involving human subjects.

In the field of biomedical research a fundamental distinction must be recognised between medical research in which the aim is essentially diagnostic or therapeutic for a patient, and medical research, the essential object of which is purely scientific and without implying direct diagnostic or therapeutic value to the person subjected to the research.

Special caution must be exercised in the conduct of research which may affect the environment, and the welfare of animals used for research must be respected.

Because it is essential that the results of laboratory experiments be applied to human beings to further scientific knowledge and to help suffering humanity, the World Medical Association has prepared the following recommendations as a guide to every physician in biomedical research involving human subjects. They should be kept under review in the future. It must be stressed that the standards as drafted are only a guide to physicians all over the world. Physicians are not relieved from criminal, civil and ethical responsibilities under the laws of their own countries.

Basic Principles

1. Biomedical research involving human subjects must conform to generally accepted scientific principles and should be based on adequately performed laboratory and animal experimentation and on a thorough knowledge of the scientific literature.
2. The design and performance of each experimental procedure involving human subjects should be clearly formulated in an experimental protocol which should be transmitted for consideration, comment and guidance to a specially appointed committee independent of the investigator and the sponsor provided that this independent committee is in conformity with the laws and regulations of the country in which the research experiment is performed.
3. Biomedical research involving human subjects should be conducted only by scientifically qualified persons and under the supervision of a clinically competent medical person. The responsibility for the human subject must always rest with a medically qualified person and never rest on the subject of the research, even though the subject has given his or her consent.
4. Biomedical research involving human subjects cannot legitimately be carried out unless the importance of the objective is in proportion to the inherent risk to the subject.
5. Every biomedical research project involving human subjects should be preceded by careful assessment of predictable risks in comparison with foreseeable benefits to the subject or to others. Concern for the interests of the subject must always prevail over the interests of science and society.
6. The right of the research subject to safeguard his or her integrity must always be respected. Every precaution should be taken to respect the privacy of the subject and to minimize the impact of the study on the subject's physical and mental integrity and on the personality of the subject.
7. Physicians should abstain from engaging in research projects involving human subjects unless they are satisfied that the hazards involved are believed to be predictable. Physicians should cease any investigation if the hazards are found to outweigh the potential benefits.
8. In publication of the results of his or her research, the physician is obliged to preserve the accuracy of the results. Reports of experimentation not in accordance with the principles laid down in this Declaration should not be accepted for publication.
9. In any research on human beings, each potential subject must be adequately informed of the aims, methods, anticipated benefits and potential hazards of the study and the discomfort it may entail. He or she should be informed that he or she is at liberty to abstain from participation in the study and that he or she is free to withdraw visor her consent to participation at any time. The physician should then obtain the subject's freely given informed consent, preferably in writing.
10. When obtaining informed consent for the research project the physician should be particularly cautious if the subject is in a dependent relationship to him or her or may consent under duress. In that case the informed consent should be obtained by a physician who is not engaged in the investigation and who is completely independent of this official relationship.
11. In case of legal incompetence, informed consent should be obtained from the legal guardian in accordance with national legislation. Where physical or mental incapacity makes it impossible to obtain informed consent, or when the subject is a minor, permission from the responsible relative replaces that of the subject in accordance with national legislation.

Whenever the minor child is in fact able to give a consent, the minor's consent must be obtained in addition to the consent of the minor's legal guardian.

1. The research protocol should always contain a statement of the ethical considerations involved and should indicate that the principles enunciated in the present Declaration are complied with.

## Appendix 2.

## Informed Consent

In compliance with the recommendations of the Somerset West revision of 1996 of the Declaration of Helsinki, each subject must be adequately informed of the aims, methods, anticipated benefits, potential hazards and the discomfort the study may entail, as well as the subject’s right to abstain from participation in the study and to withdraw at any time. The investigator should then obtain the subject’s freely given consent, in writing, before entering the patient into the trial or prior to performing any unusual or nonroutine procedure that involves risk to the patient. The person giving the consent should sign the consent form. If the patient is unable to give consent, the consent (or assent) of the patient’s legal representative must be obtained prior to entering the patient into the trial or performing any unusual or nonroutine procedures.

A copy of the informed consent document to be used will be submitted by the investigator to the IRB/EC for their review and approval prior to the start of the study. The investigator shall provide a copy of the signed informed consent to the patient and a copy will be maintained in the patient’s record file. Before initiation of the study, the investigator must provide the Sponsor with a copy of the IRB/EC approved consent form.

# INFORMED CONSENT

**TITLE OF STUDY.** Effects of Sequential Treatment Interruption: A single center, randomized, non-blinded study of the safety and efficacy of enhancing anti-HIV immunity through structured treatment interruptions (STIs) in chronically HIV-infected individuals with confirmed adherence to highly active anti-retroviral therapy (HAART)

Principal Investigator: Jay R. Kostman, MD, Philadelphia FIGHT

Co-Investigators: Karam Mounzer, MD, Philadelphia FIGHT

Paul Kernozek, MS, RN, CNS, Philadelphia FIGHT

Cecile Gallo, BSN, RN, Philadelphia FIGHT

# **Voluntary Participation in Research**

This consent form gives you information about the study that will be discussed with you. Once you understand the study and, if you agree to take part in the study, you will be asked to sign this consent form. After you sign this consent form, you will be given a copy for your personal records. Before you learn more information about this study, you should understand that your participation in the study is completely voluntary. You should understand that you may decide to not participate in the study or to withdraw from the study at any time without losing any of the benefits of your routine medical care.

# **PURPOSE OF THE STUD**Y

You have been invited to participate in this research study, to investigate the safety, immunologic, and virologic outcomes of sequential periods of interruptions of anti-HIV medications (HAART) in chronically HIV-infected persons. This study will investigate how your immune system responds once you stop your anti-HIV medications and if you can increase your ability to fight HIV-1 as a consequence of a limited period of viral replication in your body. Technically, the primary objectives of this study are to evaluate the safety of sequential *Structured Treatment Interruptions (STIs)*, to determine the effect of *STIs* on your ability to fight HIV-1 (HIV-specific cellular immunity) compared to individuals who remain under their HAART medications, and to determine if sequential interruptions in treatment as referred to as “*Structured Treatment Interruptions*” (*STIs)* result in a period of 4 weeks or more of viral suppression in the absence of therapy when comparing the consequence of an interruption in therapy between patients having completed sequential STIs to those maintaining continual viral suppression under HAART.

**DESCRIPTION OF THE PROJECT**

Therapy for HIV infection can control the levels of HIV in the blood (viral load). Even when the viral load is kept at levels that are undetectable, the immune system may not function normally. Preliminary data from an ongoing observational study of treatment interruptions performed at Philadelphia FIGHT suggests an acute viral rebound can boost resistance against HIV-1 (anti-HIV cellular immunity), one of the important functions of the immune system to control your viral load. Specifically, the viral rebound associated with interruptions in HAART medications showed a significant increase in two components of your immune response against HIV-1: anti-HIV-1 CD4 (T-helper cells of the immune system) and CD8 (T-killer cells of the immune system) responses. Data from other studies suggest that increases in these responses, anti-HIV-1 specific responses, can be associated with an increased control of plasma viral replication (viral load) in the absence of HAART.

This study is designed for people who are willing to stop their current HAART medications after their viral load has been suppressed for at least 6 months. Before entering the study, your doctor and you will discuss the possible benefits and risks of stopping your anti-HIV medications. You should know that it is currently recommended to continue HAART regimens for HIV as long as the viral load is suppressed to an acceptable level. However, people sometimes decide to stop their HIV medications because they are experiencing side effects that are related to those medications. It has been observed that viral load increases in such people after stopping their medications usually responds to restarting the same medicines.

At the beginning of the study, the research staff will confirm with you that you understand the potential risks and benefits of stopping your HAART medications. 42 subjects will be recruited through the Jonathan Lax Immune Disorders Treatment Center of Philadelphia FIGHT for participation in this study. When you start the study, you will be randomly assigned (like tossing a coin) to one of two groups. You will be assigned to either (1) remain on your current HAART medications or (2) to undergo three successive (2-week, 4-week and 6-week) treatment interruptions. If assigned to the control group, you will maintain your HAART regimen for a period of 40 weeks before interrupting your therapy for up to a >10-week period referred to as “*Comparison Treatment Interruption” (CTI).* The second group will be referred to as the “ *Structured Treatment Interruption” (STI)* group due to a series of sequential treatment interruption periods that will be completed by this group before a final interruption to be similar to the CTI period described above for the control group. Participation in the STI group will last approximately 56 weeks. You will have an equal (50%) chance of assignment to one of the two groups.

# **INCLUSION AND EXCLUSION CRITERIA**

In order to be eligible for this study, you must be HIV-positive and 18 years old or greater. You will be eligible to participate in this study if you also meet the following conditions:

- You have had a documented viral load (HIV RNA) of greater than 10,000 copies/ml prior to initiating your current HAART regimen;
- You have maintained a viral load (HIV RNA) less than 500 copies/ml while on an uninterrupted HAART regimen containing 2 nucleoside reverse transcriptase inhibitors (NRTIs) and either 1 protease inhibitor (PI) or 1 non-nucleoside reverse transcriptase inhibitor (NNRTI) for 6 months or greater;
- You have a viral load (HIV RNA) less than 50 copies/ml at screening;
- You have a CD4 count greater than 400 cells at screening and have never had a CD4 count of 100 cells or less in the past;
- If female, you have a negative pregnancy test within 2 weeks of any interruption in HAART medications and agree to use appropriate contraceptive methods while participating in the study;
- You are able to carry out “normal activities of daily living” with minimal effort;
- You are willing to abstain from immunomodulatory (directly influence the immune system) drugs while participating in the study;
- You are willing to adhere to the treatment and treatment interruption schedules approved by the study investigators in conjunction with your primary provider. This willingness will not impact on your right to withdraw from the study at any point.

You will be excluded from participation in this study if:

- You are currently pregnant or are breast feeding;
- You have been prescribed immunomodulatory (directly influence the immune system) therapy within 4 weeks of screening;
- You have a significant co-existing condition, such as low blood cells (anemia), low white blood cells (neutropenia), low platelet cells (thrombocytopenia), liver disease, kidney disease, or other conditions, such as acute drug/alcohol abuse or dependence which, in the opinion of the investigator, would interfere with study compliance.

# **PERSONAL INFORMATION**

If you agree to participate in this study, you will be asked to provide the research staff with the names of any friends or family members that the research staff may contact if they experience problems with getting in touch with you. If the information needed is not available from your medical records and you cannot be reached directly, the research staff may attempt to reach you by contacting the friends or family members who you have specified. The research staff, however, will not release any confidential information about you if it becomes necessary to contact them.

# **STUDY MEDICATIONS AND TESTING**

There are no experimental drugs involved with this study. All the medications in the various HAART regimens have been approved by the Food & Drug Administration (FDA). The cost of your HAART medications will not be provided by this study. Tests and other study procedures which are not a part of routine care and are required by this study will be provided free of charge.

# **STUDY PROCEDURES**

If you agree to participate in this study, a medical history and physical examination will be performed during screening, at Day 0 and on a monthly basis throughout the study. Your past medical history will be obtained and you will be required to provide information about HIV-related and non-HIV-related diagnoses, prior anti-HIV therapies, immunomodulatory therapies, vaccinations, current prescription medications and laboratory results pertaining to your year of diagnosis, viral loads and CD4 counts. Medical records will be reviewed to determine peak (highest) HIV-1 viral load, lowest (nadir) CD4 count, history of opportunistic infection and other information to determine eligibility for participation in this study. You will be asked to sign a release of medical records form, if necessary, to obtain this relevant medical information from sources other than the Lax Treatment Center.

# **CONTROL GROUP**

If you are randomized to the control group, you will continue your HAART medications and will be followed every 4 weeks for a period of 40 weeks (10 visits). Blood specimens will be obtained at these visits to measure complete blood count (CBC), viral load (HIV RNA), CD4 cell counts and other tests to measure the function of your immune system. A maximum of 6 tablespoons (up to 90 ml) of blood will be obtained at any one visit with a minimum of one and a half tablespoons (22.5 ml) per visit. Any changes in your clinical status, as well as changes in your antiretroviral medications, will be recorded. If your viral load (HIV RNA) goes above 1,000 copies/ml for more than two visits. If you maintain a viral load less than 50 copies/ml, you will have your HAART regimen stopped within a week after the Week 40 visit. This withdrawal of treatment is called a *Comparison Treatment Interruption (CTI)*. During this *Comparison Treatment Interruption (CTI)* period, you will be monitored at weekly visits to evaluate any clinical changes. You will have blood specimens drawn to measure complete blood count (CBC), viral load (HIV RNA), CD4 cell counts and other tests to measure the function of your immune system. You will restart your HAART regimen after your viral count is greater than 30,000 copies/ml for 3 consecutive weeks without any evidence of a decrease greater than 70% (0.5 log) between measurements. A final viral load (HIV RNA) will be obtained 6 weeks after you restart your HAART medications. If your viral load shows less than a 70% (0.5 log) reduction from your peak (highest) viral load, GART (a type of HIV resistance) testing will be offered to assist your primary care provider in making any necessary changes in your HAART medications. If you decide to discontinue your participation or are withdrawn prior to completion of the study, you will be asked to come in for a termination visit.

# **EXPERIMENTAL GROUP**

If you are randomized to the sequential *Structured Treatment Interruption (STI)* group, you will have your HAART regimen interrupted up to 4 sequential times according to the following guidelines. During all periods prior to the fourth interruption of therapy which represents the *Comparison Treatment Interruption (CTI)*, you will be monitored every 2 weeks for any changes in clinical status, complete blood count (CBC), viral load (HIV RNA), CD4 cell counts and other tests to measure the function of your immune system. A maximum of 6 tablespoons (up to 90 ml) of blood will be obtained at any one visit with a minimum of one and a half tablespoons (22.5 ml) per visit.

## Priming Structured Treatment Interruption (STI)

Within 4 weeks of randomization and baseline blood specimens, your HAART regimen will be interrupted for 2 weeks. After 14 days without therapy, you will restart and continue your HAART regimen until 2 viral load (HIV RNA) measurements less than 50 copies/ml. If you do not show greater than a 70% reduction (0.5 log) in your peak (highest) viral load by 6 weeks after you restart your HAART medications, or you don’t show a viral load (HIV RNA) less than 50 copies/ml by 20 weeks after you restart your HAART medications, you will be offered GART (a type of HIV resistance) testing to assist your primary care provider in making changes in your HAART medications. This first *Structured Treatment Interruption (STI)* period is called the *Priming Structured Treatment Interruption (Priming STI*). You will be withdrawn from the study if you do not achieve a viral load of less than 50 copies/ml by 20 weeks after you restart your HAART medications during the *Priming STI*.

*CD4 Boost Structured Treatment Interruption (STI)*

Within a week after 2 viral load (HIV RNA) measurements less than 50 copies/ml during the *Priming STI* period, your HAART regimen will be withdrawn a second time for a period of 4 weeks. After 28 days without therapy, you will restart and continue your HAART regimen until 2 successive viral load (HIV RNA) measurements taken 2 weeks apart are less than 50 copies/ml. If you do not show greater than a 70% reduction (0.5 log) in your peak (highest) viral load by 6 weeks after you restart your HAART medications, or you don’t show a viral load (HIV RNA) less than 50 copies/ml by 20 weeks after you restart your HAART medications, you will be offered a test to assess HIV resistance to your medications (GART test) to assist your primary care provider in making changes in your HAART medications. This second *Structured Treatment Interruption (STI)* period is called the *CD4 Boost Structured Treatment Interruption (CD4 Boost STI*). You will be withdrawn from the study if you do not achieve a viral load of less than 50 copies/ml by 20 weeks after you restart your HAART medications during the *CD4 Boost STI*.

*CD8 Boost Structured Treatment Interruption (STI)*

Within a week after 2 viral load (HIV RNA) measurements less than 50 copies/ml during the *CD4 Boost STI* period, your HAART regimen will be withdrawn a third time for a period of 6 weeks. After 42 days without therapy, you will restart and continue your HAART regimen until 2 successive viral load (HIV RNA) measurements taken 2 weeks apart are less than 50 copies/ml. If you do not show greater than a 70% (0.5 log) reduction in your peak (highest) viral load by 6 weeks after you restart your HAART medications, or you don’t show a viral load (HIV RNA) less than 50copies/ml by 20 weeks after you restart your HAART medications, you will be offered a test to assess HIV resistance to your medications (GART test) to assist your primary care provider in making changes in your HAART medications. This third *Structured Treatment Interruption (STI)* period is called the *CD8 Boost Structured Treatment Interruption (CD8 Boost STI*). You will be withdrawn from the study if you do not achieve a viral load of less than 50 copies/ml by 20 weeks after you restart your HAART medications during the *CD8 Boost STI*.

*Comparison Treatment Interruption (CTI)*

Within a week after 2 viral load (HIV RNA) measurements less than 50 copies/ml during the *CD8 Boost STI* period, your HAART regimen will be withdrawn a final time. You will be monitored weekly for up to 10 weeks to evaluate clinical changes. You will have blood specimens drawn to measure complete blood count (CBC), viral load (HIV RNA), CD4 cell counts and other tests to measure the function of your immune system. You will restart your HAART regimen after your viral load is greater than 30000 copies/ml for 3 consecutive weeks without any evidence of a decrease greater than 70% (0.5 log) between measurements. A final viral load (HIV RNA) will be obtained 6 weeks after you restart your HAART medications. If you do not show greater than a 70% reduction (0.5 log) in your peak (highest) viral load by 6 weeks after you restart your HAART medications, or you don’t show a viral load (HIV RNA) less than 50 copies/ml by 20 weeks after you restart your HAART medication, you will be offered a test to assess HIV resistance to your medications (GART test) to assist your primary care provider in making changes in your HAART medications. If you decide to discontinue your participation or are withdrawn prior to completion of the study, you will be asked to come in for a termination visit.

**RISKS AND DISCOMFORTS**

The risks of having blood drawn include pain, inflammation (swelling that is painful, red and warm), bruising at the point where the needle enters the skin, and, rarely, fainting or infection.

The potential risks of interrupting HAART medications in a structured fashion are not completely known at this time. The current recommendation for treatment of your HIV-infection is maintenance of your HAART treatment for life as long as it can achieve adequate suppression of viral replication and not induce life-threatening complications. Although preliminary data from an ongoing observational study at Philadelphia FIGHT and other clinical research sites do not suggest that complete interruption in HAART induces immediate or irreversible risks to patients, interruptions in HAART could diminish your ability to suppress the viral load (HIV RNA) in your body, lower your CD4 cell counts, weaken the ability of your HAART regimen to fight the virus in your body (viral resistance) or result in progression of your HIV disease.

The drugs used in HAART regimens can interact with other medications. These drug interactions may change the effect of the HAART medications or other medications on the body. There is a possible risk of serious or life-threatening side effects when other medications are taken with your HAART medications. Side effects from your HAART medications and other medications could show up at any time during your participation in this study. If you experience any side effects or other symptoms between your scheduled study visits, you should report them immediately to your research doctor or research nurse. Your doctor may want to adjust the dose or dosing schedule of your HAART medications or may ask you to stop taking the medications. The side effects listed below are the most common or most serious seen with the different drug classes. You should contact your study nurse or study doctor if you have any questions about any additional side effects you may experience during your participation in this study.

*Nucleoside Reverse Transcriptase Inhibitors (NRTIs)*

This class of drugs can result in abnormal levels of acid in your blood (lactic acidosis) and affect liver function (severe hepatomegaly or enlarged liver) increasing the amount of fat in the liver (steatosis or fatty liver) that may result in liver failure. Other complications and death have been reported with the use of nucleoside reverse transcriptase inhibitors (NRTIs) alone or in combination. This class of anti-HIV drugs includes ZDV (AZT, Zidovudine or Retrivir™), ddI (Didanosine or Videx™), ddC (Zalcitabine, or Hivid™), 3TC (Lamivudine or Epivir™), d4T (Stavudine or Zerit™) and ABC (Abacavir or Ziagen™).

*Non-Nucleoside Reverse Transcriptase Inhibitors (NNRTIs)*

Another class of drugs which inhibits reverse transcriptase in the life cycle of HIV is commonly referred to as the “non-nukes”. Many patients experience a mild rash upon starting therapy. Other side effects include fever, muscle soreness, body ache, headache, light-headedness, insomnia, abnormal dreams, nausea, diarrhea and elevated liver function. In very rare cases, a potentially life-threatening skin condition known as Stevens-Johnson syndrome has been reported. This class of anti-HIV drugs includes NVP (Nevirapine or Viramune™), DLV (Delavirdine or Rescriptor™) and EFV (DMP-266, Efavirenz or Sustiva™).

*Protease Inhibitors (PIs)*

The use of potent antiretroviral drug combinations, such as HAART, usually includes a protease inhibitor. This class of anti-HIV drugs includes AMP (Amprenavir or Agenerase™), IDV (Indinavir or Crixivan™), NFV (Nelfinavir or Viracept™), RTV (Ritonavir or Norvir™), and SQV (Saquinavir or Fortovase™). Protease inhibitors (PIs) have been associated with an abnormal distribution (placement) of body fat and wasting (thin appearance). Some of these body changes include an increase in fat around the waist and stomach area, increase in fat on the back of the neck, breast enlargement and thinning of the face, legs and arms. The use of HAART regimens that commonly contain a PI has also been associated with changes in fat metabolism including elevated triglycerides (increased fatty acid in the blood) and/or elevated cholesterol. The use of protease inhibitors may be associated with the development or worsening of elevations in blood sugar and diabetes. There have been reports of increased bleeding in HIV-infected persons with bleeding disorders (hemophilia) who were treated with PIs. It is not known if PIs were the cause of these bleeding episodes.

# **NEW INFORMATION**

You will be informed of any new information learned during the study that might cause you to change your mind about staying in the study. At the end of the study, you will be told when the study results may be available and how to learn about them.

# **BENEFITS**

Participating in this study and taking your HAART medications as directed may slow your HIV infection, but no guarantee can be made. Since the results of viral load tests and CD4 counts will be available, you and your health care provider will have additional information to make decisions about the management of your HIV disease. You may receive no benefit from your participation in this study. Knowledge gained from your participation in this study, however, may help others who have HIV infection. You may contribute to the understanding of how the immune system responds in HIV-positive individuals who have taken HAART medications and achieved viral suppression (low levels of viral load), as well as how the immune system responds when treatment is interrupted.

**ALTERNATIVES**

You should understand that if you do not wish to participate in this study, there are alternative therapies available to you for the treatment of your condition. If you decide not to participate, withdraw your participation, or if you are discontinued from this study, your doctor and/or research staff will discuss other treatment options with you. You should feel free to discuss the disease and various treatment options with your doctor and/or research staff at any time.

**CONFIDENTIALITY**

You have a right to privacy and all information obtained in this study that can be identified with your name will remain confidential as far as possible within state and federal law. Your name will not be revealed in any reports, any communication between investigators associated with Philadelphia FIGHT in regards to this study (other than Jay Kostman, your provider and the study nurse) or publications resulting from this study. The FDA, members of the medical review board or ethics committee, representatives of Philadelphia FIGHT and their designees, and representatives of other health or research authorities (The Wistar Institute, The Aaron Diamond Research Center, The Gladstone Institute, The University of Pennsylvania) may inspect and copy information from your medical records which relate to your participation in this study in order to verify study procedures and data to the extent such inspection is permitted by federal and local laws and regulations. These records will not be made publicly available. Samples of your blood obtained in the course of your participation on this study will be distributed among investigators in charge of the analysis proposed in the study (The Wistar Institute, The Aaron Diamond Research Centers, the Gladstone Institute, The University of Pennsylvania). Your identity will not be disclosed to any investigator on this study other than Dr. Jay Kostman. The results of this study will be reported to the scientific community and other health authorities. If you become pregnant while on this study, Philadelphia FIGHT will require access to your and/or your infant’s clinic/hospital records during pregnancy and for at least 8 weeks following delivery, if applicable. By signing this informed consent form, you are authorizing such access to your medical records.

**WITHDRAWAL FROM THE STUDY**

You understand that you may decide not to participate or may withdraw from this study at any time without penalty or loss of benefits to which you are otherwise entitled. Your participation in this research study is entirely voluntary. Your treatment by and relations with the physicians and organizations involved in this study will not be affected now or in the future. Your participation in the study may be terminated by the investigator for any of the following reasons:

- You require additional medications which may interfere or interact with any of the study medications;
- You experience serious side effects associated with any of the study drugs;
- You do not take the study medications as prescribed and are unwilling to cooperate with the study procedures;
- You become pregnant;
- Philadelphia FIGHT terminates the study;

**COMPENSATION**

If you are randomized to the control group (no more than 21 visits), you will receive up to a maximum compensation of $525.00 for your participation in this study. You will receive $15.00 at the end of each study visit. When you complete the study or are withdrawn under the conditions described in the study procedures section, you will receive a final payment (not to exceed $210.00) of $10.00 for each completed study visit. If you are randomized to the *Structured Treatment Interruption (STI)* group (no more than 36 visits), you will receive up to a maximum compensation of $900.00 for your participation in this study. You will receive $15.00 at the end of each study visit. When you complete the study or are withdrawn under the conditions described in the study procedures section, you will receive a final payment (not to exceed $360.00) of $10.00 for each completed study visit.

**RESEARCH-RELATED INJURY**

If you are injured because of being in this study, Philadelphia FIGHT will give you immediate necessary treatment for your injuries. The cost of this treatment will be charged to you, your insurance company or other third party payer. You will then be told where you may receive additional treatment for injuries. There is no program for monetary compensation or other forms of compensation for such injuries. You should understand that you have not waived any of the legal rights that you would otherwise have as a participant in any investigational study.

**RESEARCH SUBJECT’S RIGHTS & CONTACT INFORMATION**

Before giving your consent by signing this form, you are required to have read it and have been fully informed of the methods and means of administration of the study medications being used and the side effects that might occur from the use of these study medications. You are required to have spoken directly to the study physician, Dr. Kostman, or his designate, who has answered to your satisfaction all of your questions concerning this study in language that you can understand.

- If you have any questions on the protocol, please contact Dr. Jay Kostman at 215-985-4448.
- If you have any questions regarding your rights as a subject participating in this research study, you may contact Ronda Goldfein, the Chairperson of the Institutional Review Board for Philadelphia FIGHT at 215-587-9377.

**CONSENT**

In signing this consent form, you are freely agreeing to participate in this study and you acknowledge that no study-related procedures have been performed prior to you signing this consent form. You also acknowledge that you have received a copy of this form.

______________________________

Subject’s Printed Name

______________________________ _______________

Signature of Subject Date

______________________________ _______________

Signature of Witness Date

______________________________ _______________

Signature of Investigator Date

**Addendum for Women of Child-Bearing Potential**

**If not applicable, check here .**

A member of the research staff, _________________________, has reviewed information on pregnancy prevention for women of childbearing potential who are participating in clinical trials. You understand that you will be receiving anti-HIV medications in your HAART regimen for which there may be side effects and discomforts to you and the embryo or fetus which are not yet known. Therefore, women of childbearing age and ability (that is, not surgically sterile or post-menopausal) and men who consent to participate in this study must practice effective birth control including a barrier method (that is, a condom). You should not become pregnant while you are participating in this study. You should understand that you should immediately call Dr. Jay Kostman, his designee or the study coordinator at 215-985-4448 if :

- You are pregnant or you think you may be pregnant;
- You have missed your period or it is late, or you have a change in your usual menstrual cycle (for example, heavier bleeding during your period or bleeding between periods);
- You have changed or plan to change your birth control method, or you need to take any prescription drug or other medication not given to you by Dr Jay Kostman or his designee.

If you become pregnant during the course of the study, you will be withdrawn from the study and referred for obstetrical (pregnancy) health care. All financial aspects of obstetrical, child or related health care are your responsibility.

______________________________

Subject’s Printed Name

______________________________ _______________

Signature of Subject Date

______________________________ _______________

Signature of Witness Date

______________________________ _______________

Signature of Investigator Date

**Appendix 3.**

## Performance Index

|  |  |
| --- | --- |
| **Karnofsky Scale** | **(%)** |
|  |  |
| **Normal, no complaint.** | **100** |
| **Able to carry on normal activities; minor signs or symptoms of disease.** | **90** |
| **Normal activity with effort.** | **80** |
| **Cares for self. Unable to carry on normal activity or to do active work.** | **70** |
| **Ambulatory. Requires some assistance but able to care for most of own needs.** | **60** |
| **Requires considerable assistance and frequent medical care.** | **50** |
| **Disabled; requires special care and assistance.** | **40** |
| **Severely disabled, hospitalization indicated though death not imminent.** | **30** |
| **Very sick. Hospitalization necessary. Active supportive treatment necessary.** | **20** |
| **Moribund.** | **10** |
| **Dead.** | **0** |

**Appendix 4 – Case Report Forms**

**Appendix 5 Study Flow Charts**

**Appendix 6 Patient Monitoring Flowsheets**
