# Supplementary material for: Randomized, Controlled Trial of Therapy Interruption in Chronic HIV-1 Infection
Source: PLoS Med. 2004 Dec 28;1(3):e64. doi: 10.1371/journal.pmed.0010064 (PMC539050; doi:10.1371/journal.pmed.0010064)
Supplement: Table S1 — (36 KB DOC). [file pmed.0010064.st001.doc]

**Table S1.** Subjects with Detected Resistance during Phase II: Regimen at Initiation of Phase II and Subsequent Post-Study Follow-up to August 2004

**Patient Group Drug Regimen Weeks of Drug Regimen Reason for Weeks to <50*** **Weeks of Follow-up Reason For Current Drug**

**Start of Phase II** **Phase II** **At End of Phase II Regimen Change After Therapy with <50*** **On Subsequent Regimen**

**Re-Initiation Same Regimen Regimen Change**

S13 C.T./S.I. IDV, RTV, ZDV, 3TC 38 Stayed Off ART Not Applicable Not Applicable Not Applicable Not Applicable Not Applicable

S32 C.T./S.I. d4T, 3TC, EFZ 35 d4T, 3TC, EFZ No Change 10 Interrupted ART at <50* Not Applicable Not Applicable

S40 C.T./S.I. IDV, RTV, d4T 8 LOP, RTV, ddI, TNV P.-P.P.D.S.E. 25 Not Available No Change LOP, RTV, ddI, TNV

S4 R.I. IDV, RTV, d4T, 3TC 32 IDV, RTV, d4T, 3TC No Change 11 2 P.-P.P.D.S.E. TNV, 3TC, EFZ

S7 R.I. IDV, RTV, d4T, 3TC 31 TNV, 3TC, EFZ, NVP P.-P.P.D.S.E. 6 55 P.-P.P.D.S.E. TNV, 3TC, NVP

S19 R.I. ddI, 3TC, EFZ 19 ddI, 3TC, EFZ No Change 15 56 Concern for RTV, ATV, TNV, 3TC

Potential Resistance to EFZ†

S22 R.I. NLF, ddI, NVP 38 NLF, ddI, NVP No Change 10 42 No Change NLF, ddI, NVP

S23 R.I. ddI, d4T, EFZ 14 ddI, d4T, EFZ No Change 11 53 P.-P.P.D.S.E. RTV, ATV, TNV, 3TC

S35§ R.I. LOP, RTV, ABV, EFZ 27 LOP, RTV, ABV, TNV P.-P.P.D.S.E. 6 4 P.-P.P.D.S.E. RTV, ATV, ABV, 3TC

S43 R.I. ddI, d4T, EFZ 30 ddI, d4T, EFZ No Change 9 24 P.-P.P.D.S.E. TNV, ddI, EFZ

S45¶ R.I. NLF, d4T, 3TC¶ No open-Ended TI Not Applicable Not Applicable Not Applicable Not Available Not Available Not Available

S51 R.I. NLF, d4T, 3TC 28 NLF, d4T, 3TC No Change 6 11 P.-P.P.D.S.E. NLF, TNV, 3TC

Abbreviations: C.T./S.I., Continuous Therapy/Single Interruption; R.I., Repeated Interruptions Group; ART, Antiretroviral Therapy; P.-P.P.D.S.E., Patient-Physician Preference due to Drug Side Effects; ddI, Didanosine; d4T, Stavudine; 3TC, Lamivudine; EFZ, Efavirenz; NVP, Nevirapine; ABV, Abacavir; IDV Indinavir; RTV, Ritonavir; ZDV, Zidovudine; NLF, Nelfinavir; LOP, Lopinavir; TNV, Tenofovir; ATV, Atazanavir.

*<50 copies/ml

†Regimen was changed by physician due to history of detection of K103N during Phase II even if EFZ-based regimen was still maintaining suppression at <50 copies/ml.
