# Supplementary material for: Randomized, Controlled Trial of Therapy Interruption in Chronic HIV-1 Infection
Source: PLoS Med. 2004 Dec 28;1(3):e64. doi: 10.1371/journal.pmed.0010064 (PMC539050; doi:10.1371/journal.pmed.0010064)
Supplement: Protocol S2 — Current IRB approval for study at clinical site. (179 KB PDF). [file pmed.0010064.sd002.pdf]

PHILADELPHIA
FIGHT

INSTITUTIONAL
REVIEW BOARD

Chair

Ronda Goldfein JD

Members

Will Gee
Alison Gerig
Janette Pierce
Robert Swenson MD
Robert Winn MD
Craig Wynne MD

Regulatory Associate

Brad Dunville

June 2, 2004

Jay Kostman, MD
Philadelphia FIGHT
1233 Locust St., 5th Floor
Philadelphia PA 19107

Dear Dr. Kostman,

On May 26, 2004 the Philadelphia FIGHT Institutional Review Board performed a six-month protocol progress report review of the study entitled:

"Effects of Sequential Treatment Interruption: A single center, randomized, non-blinded study of the safety and efficacy of enhancing anti-HIV immunity through structured treatment interruptions (STIs) in chronically HIV-infected individuals with confirmed adherence to highly active-anti-retroviral therapy" (Wistar STI)

This study was initially approved on February 17, 2000. The study has been reviewed by the FIGHT IRB on a monthly basis since the beginning of the study, on a quarterly basis since October 24 of 2001, and on a six-month basis since February 7 of 2003. Amendment 1 was approved on September 27, 2000. Amendment 2 was approved July 6, 2001. Demographics for study subjects and serious adverse events were reviewed and study re-approval was granted.

You are authorized to continue this study. Based on the board's evaluation of risk to human subjects and safety-related considerations, it was determined that six-month protocol progress reports will continue to be required for this study. The next six-month review will be due by November 26, 2004.

If you wish to change any aspect of this study such as the procedures, the consent form, or the investigators, please communicate your requested changes in writing to me. Changes cannot be initiated until IRB approval has been given.

Sincerely,

Ronda Goldfein, JD
Chair, Philadelphia FIGHT IRB

COPY
6.7.04
[illegible]
