# Supplementary material for: Randomized, Controlled Trial of Therapy Interruption in Chronic HIV-1 Infection
Source: PLoS Med. 2004 Dec 28;1(3):e64. doi: 10.1371/journal.pmed.0010064 (PMC539050; doi:10.1371/journal.pmed.0010064)
Supplement: Protocol S3 — IRB approval to receive study biological material at the Wistar Institute for research. (201 KB PDF). [file pmed.0010064.sd003.pdf]

THE WISTAR INSTITUTE

Office of Research Administration and Resources
Institutional Review Board

March 2, 2004

Luis Montaner, DVM, PhD
The Wistar Institute
3601 Spruce Street, Room 480
Philadelphia, PA 19104

Re: "Immune and Viral Outcomes of HIV-1 Treatment Interruption"
Human Subjects Protocol #2303192-2

Dear Dr. Montaner:

Your request to perform work on the above-referenced protocol was reviewed and approved for the use of **Blood Samples** to be obtained from Philadelphia FIGHT by **Expedited Review** of the Institutional Review Board on February 18, 2004. This approval will expire on February 17, 2005.

Please note the following information:

Adverse Events: Any injuries or other unanticipated problems involving risks to research subjects and others resulting from this study must be reported promptly to the Office of Research Administration and Resources. If the problem is serious, approval may be withdrawn pending further IRB review. **Changes or Amendments:** Proposed changes in previously approved human subject research activities must be promptly reported to the Office of Research Administration and Resources. The proposed changes may not be initiated without IRB review and approval, except where necessary to eliminate apparent immediate hazards to subjects. **Reapproval and Progress Reports:** Institutional policy and federal regulations require a timely response to requests for submission of reapproval documents and reports of progress. These requests will be sent to you annually or more frequently if required by the IRB. **Completion of Study:** Please notify the Office of Research Administration and Resources as soon as the research has been completed.

If you have any questions, please contact the Office of Research Administration and Resources, (215) 898-3708. Thank you for your excellent cooperation with the IRB.

Sincerely,

Steven M. Albelda, M.D.
Chair, Wistar IRB

SMA/afw
